# Supplementary material for: Natural genetic variation determines microglia heterogeneity in wild-derived mouse models of Alzheimer’s disease
Source: Cell Rep. Author manuscript; Available in PMC 2021 Mar 7. (PMC7937391; doi:10.1016/j.celrep.2021.108739)
Supplement: 1 [file NIHMS1672405-supplement-1.pdf]

Cell Reports, Volume *34*

Supplemental Information

Natural genetic variation determines microglia heterogeneity in wild-derived mouse models of Alzheimer's disease

Hongtian Stanley Yang, Kristen D. Onos, Kwangbom Choi, Kelly J. Keezer, Daniel A. Skelly, Gregory W. Carter, and Gareth R. Howell

Figure S1

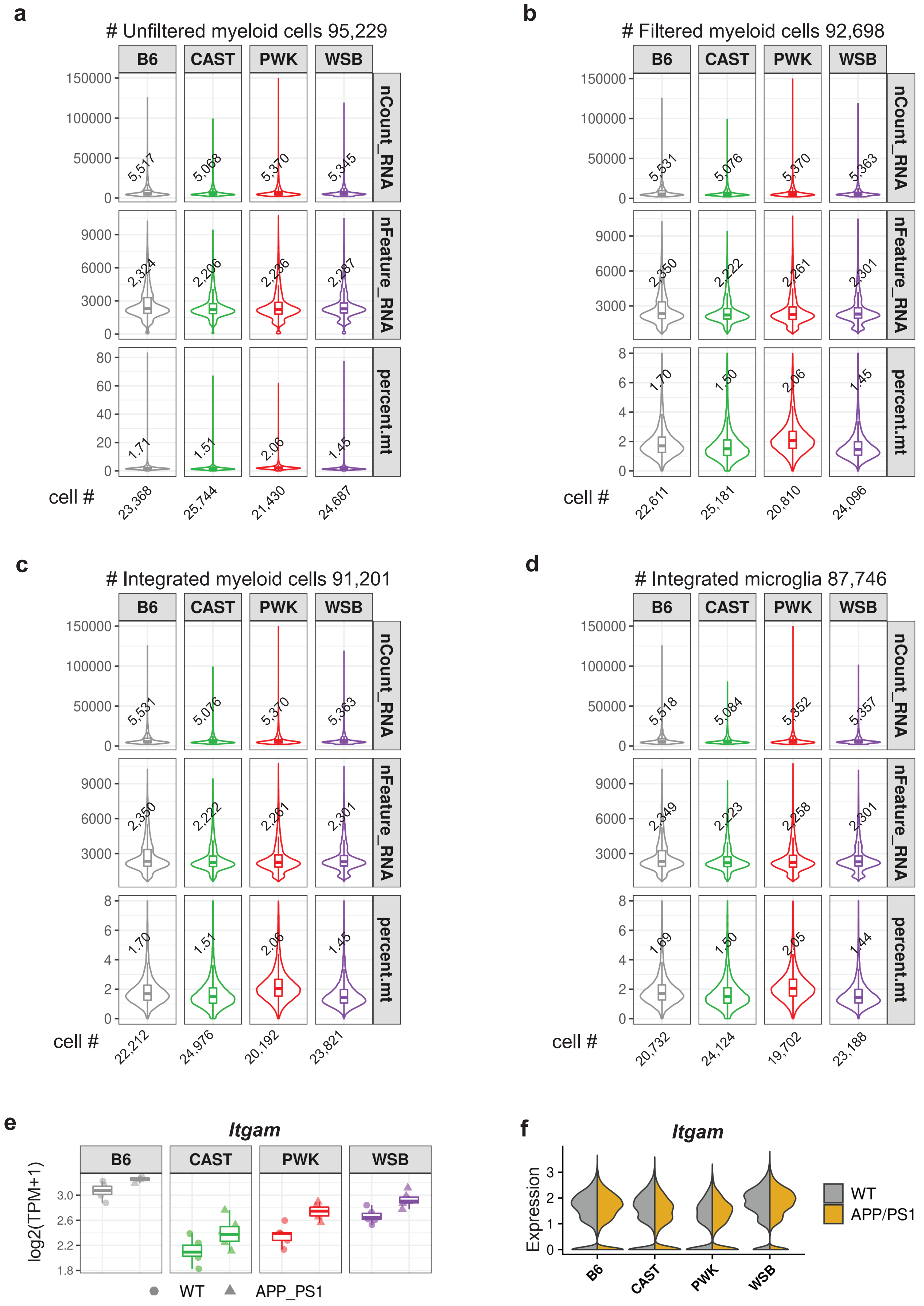
a
Unfiltered myeloid cells 95,229
B6
CAST
PWK
WSB
nCount_RNA
nFeature_RNA
percent.mt
150000
100000
50000
0
9000
6000
3000
0
80
60
40
20
0
5,517
5,068
5,370
5,345
2,324
2,206
2,236
2,287
1.71
1.51
2.06
1.45
cell #
23,368
25,744
21,430
24,687
b
Filtered myeloid cells 92,698
5,531
5,076
5,370
5,363
2,350
2,222
2,261
2,301
1.70
1.50
2.06
1.45
8
6
4
2
cell #
22,611
25,181
20,810
24,096
c
Integrated myeloid cells 91,201
5,531
5,076
5,370
5,363
2,350
2,222
2,261
2,301
1.70
1.51
2.06
1.45
cell #
22,212
24,976
20,192
23,821
d
Integrated microglia 87,746
5,518
5,084
5,352
5,357
2,349
2,223
2,258
2,301
1.69
1.50
2.05
1.44
cell #
20,732
24,124
19,702
23,188
e
Itgam
log2(TPM+1)
3.0
2.6
2.2
1.8
WT
APP_PS1
f
Itgam
Expression
3
2
1
0
WT
APP/PS1

Fig. S1 | Quality control for single-cell RNA-seq, Related to Fig.1. a-d, Violin-box plots showing the distribution of the number of total RNA reads (nCount_RNA), the total number of genes detected (nFeature_RNA) and the percent mitochondrial genes in individual cells from each strain after each quality control steps. The medians were labeled for each plot. **a**, Recovered cells after gene expression quantification by emaze-zero. **b**, Remaining cells after removing cells with high-percent mitochondrial genes (>8%). **c**, Remaining cells after myeloid cell integration based on strain. Cells that were not integrated likely did not contain sufficient anchor features as required by the *FindTransferAnchors* and *Transferdata* functions in the Seurat package. **d**, Remaining cells after microglia integration based on strain. **e**. Box plots showing *Itgam* expression detected from bulk RNA-seq from whole brain of age- and sex-matched (8 month female) mice for each strain and genotype[6].The expression of *Itgam* is significantly affected by strain (FDR= 3.3×10^{-13} , generalized linear regression) but not genotype (*APP/PS1*). **f**. Violin plots showing *Itgam* expression in all myeloid cells after integration (c) for each strain and genotype. The expression of *Itgam* was significantly affected by strain (FDR<0.05, edgeR, wild vs B6) but not genotype.

Figure S2

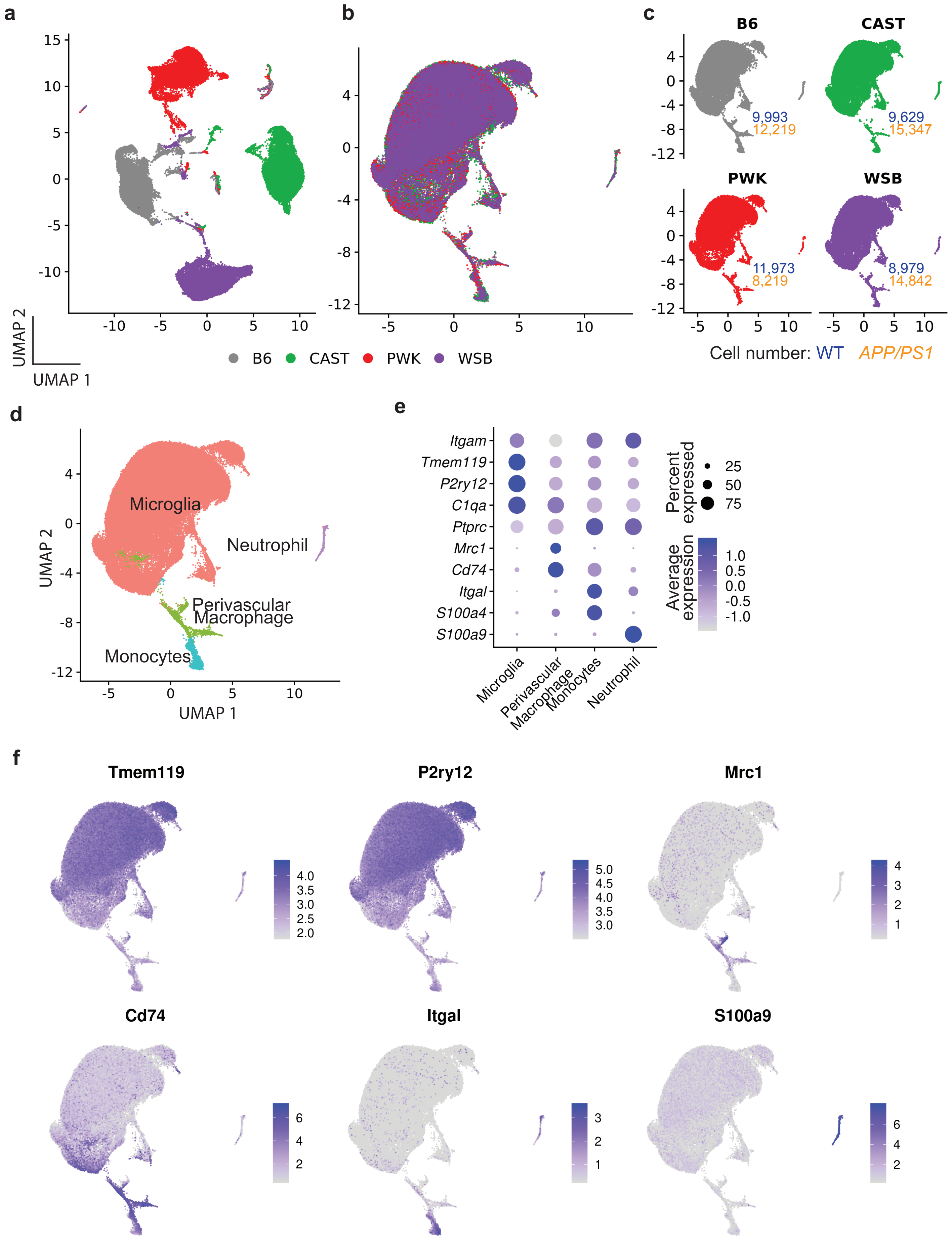

a
b
c
B6
CAST
PWK
WSB
9,993
12,219
9,629
15,347
11,973
8,219
8,979
14,842
Cell number: WT APP/PS1
UMAP 1
UMAP 2
B6 CAST PWK WSB
d
Microglia
Neutrophil
Perivascular Macrophage
Monocytes
e
Itgam
Tmem119
P2ry12
C1qa
Ptprc
Mrc1
Cd74
Itgal
S100a4
S100a9
Microglia
Perivascular Macrophage
Monocytes
Neutrophil
Percent expressed
25
50
75
Average expression
1.0
0.5
0.0
-0.5
-1.0
f
Tmem119
P2ry12
Mrc1
Cd74
Itgal
S100a9

Fig. S2 | A single-cell atlas revealed natural genetic variation shapes the transcriptome of brain myeloid cells, Related to Fig.1. a, Raw cell map of brain myeloid cells in WT and *APP/PS1* of B6, CAST, PWK and WSB mice. UMAP of 92,698 single brain myeloid cell profiles in all groups of mice (n = 3-4/genotype/strain, 29 mice in total), without data integration on the strain. **b**-**c**, Strain-integrated cell map of brain myeloid cells of WT and *APP/PS1* of B6, CAST, PWK and WSB mice. UMAP of 91,201 single brain myeloid cell profiles in all groups of mice with merged view (b) and split views based on strain (c), with canonical correlation analysis (CCA)-based integration on strains. **d**, Single-cell profiling revealed 4 major distinct myeloid cell types including microglia, perivascular macrophages, monocytes and neutrophils. The integrated UMAP showing 91,201 single brain myeloid cell profiles in all groups (29 mice) colored by myeloid cell types. **e**, Dot plot showing the classical marker genes for myeloid cell types with their percent expressed and average expression. **f**, UMAP plots featuring cluster-defining marker genes including *Tmem119*, *P2ry12*, *Mrc1*, *Cd74*, *Itgal* and *S100a9*.

Figure S3

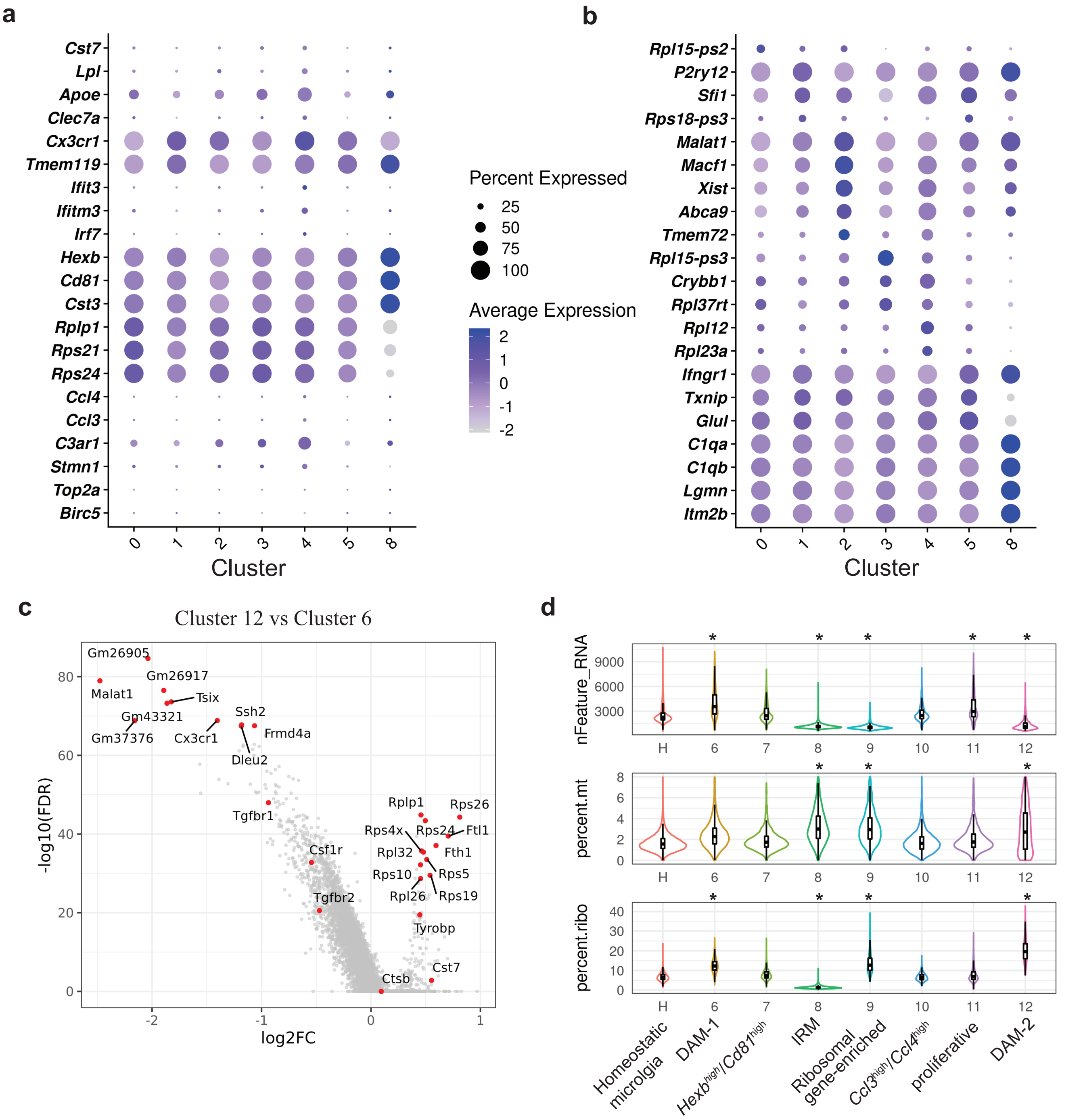

Fig. S3 | Additional features of microglia subtypes, Related to Fig.2. a, Dot plot showing the expression of top microglia marker genes of all clusters (Fig. 1b) in homeostatic microglia subtypes (clusters 0, 1, 2, 3, 4, 5) and *Hexb*^{high}/*Cd81*^{high} microglia (cluster 8). **b**, Dot plot showing the expression of the top microglia marker genes in homeostatic microglia (clusters 0,1, 2, 3, 4, 5) and 8. **c**, Volcano plot showing the top up- and down- regulated genes comparing DAM cluster 12 to DAM cluster 6. Cluster 12 showed increased expression in ribosomal genes and *Tyrobp* (log2FC > 0) and decreased expression in homeostatic microglia genes including *Cx3cr1*, *Tgfbr1*, *Csf1r* and *Tgfbr2* ($\log_2$FC < 0). **d**, Violin-box plots showing the distribution of the total number of genes detected (nFeature_RNA), the percent of mitochondrial (percent.mt) and ribosomal (percent.ribo) genes in cells from all identified microglia subtypes. * adjusted p value (p.adj) < 0.05. All comparisons were made by comparing the medians in clusters 6, 7, 8, 9, 10, 11 and 12 to combined homeostatic microglia (cluster 0 to 5), respectively, using one-way ANOVA followed by Tukey's post hoc test.

Figure S4

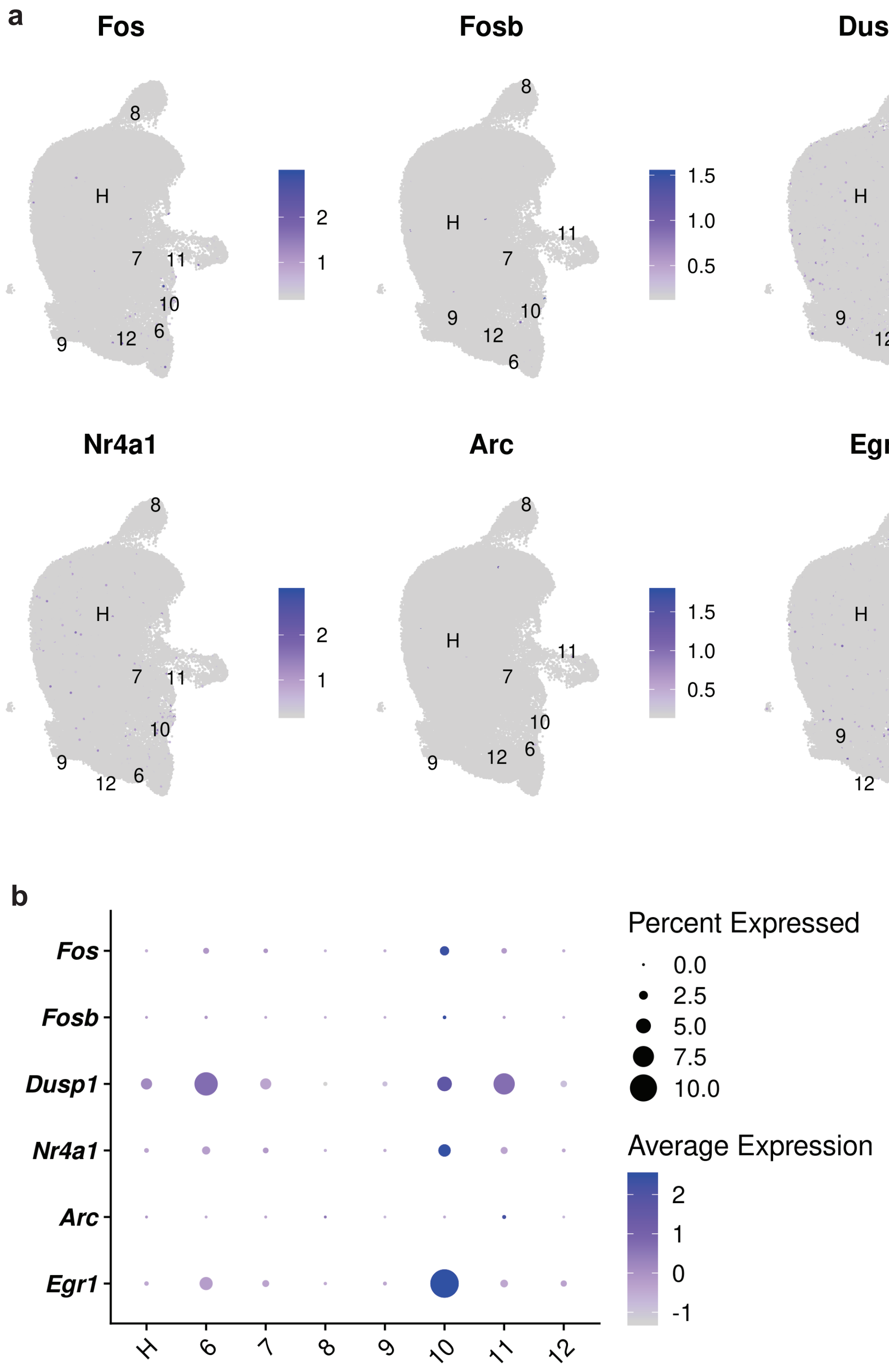

Fig. S4 | Immediate early gene expression in microglia subclusters, Related to Fig.1. a-b, UMAP plots (a) and dot plot (b) showing the expression of several common immediate early genes including *Fos*, *Fosb*, *Dusp1*, *Nr4a1*, *Arc* and *Egr1* in integrated microglia clusters (Fig. 1b).

Figure S5

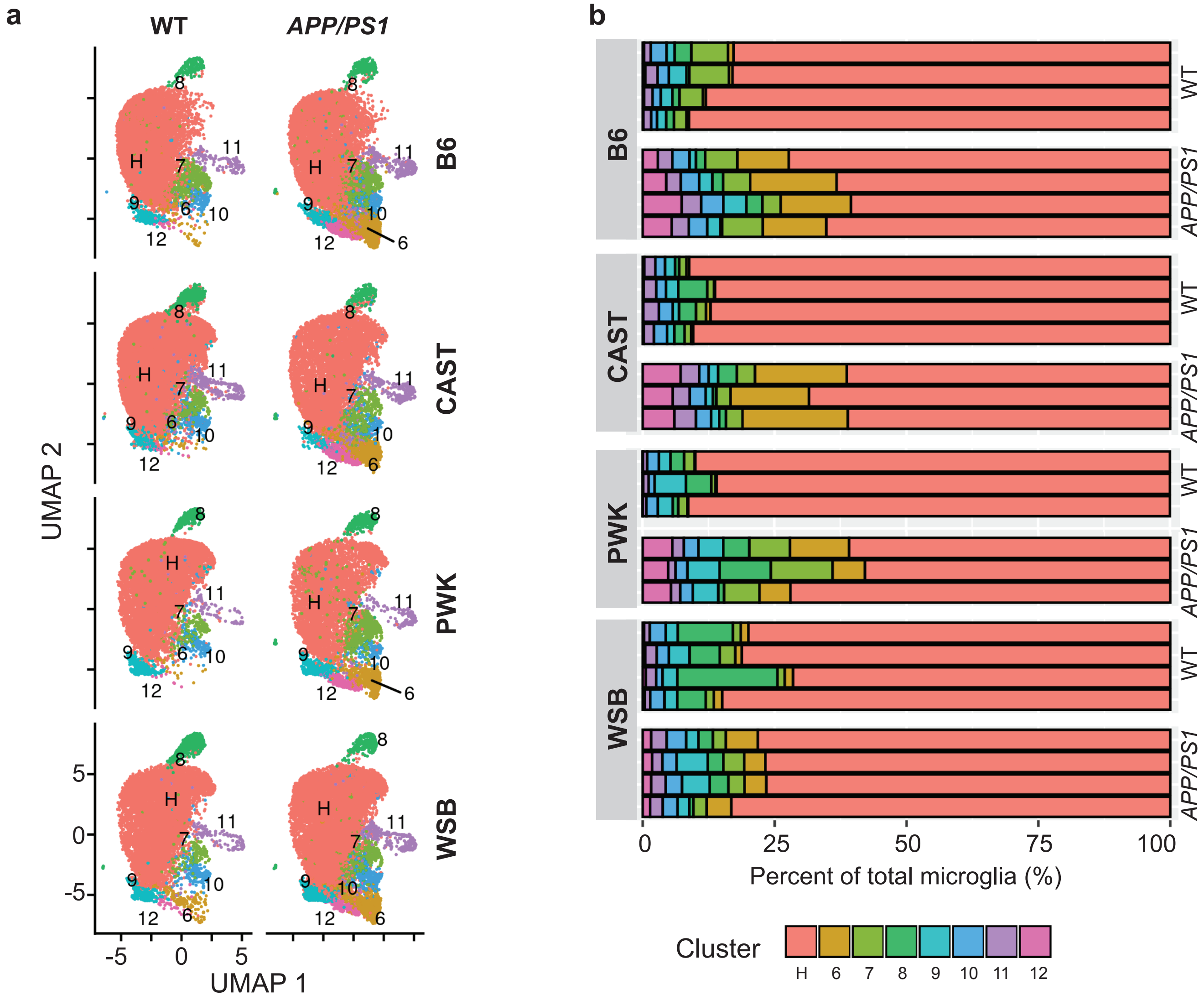

Fig. S5 | Variation in the percent of microglia subtype across strain and genotype, Related to Fig.2. **a**, UMAP plot showing microglia clusters in each strain and genotype. **b**, The percent of 8 microglia subtypes in each replicate of each strain and genotype.

Figure S6

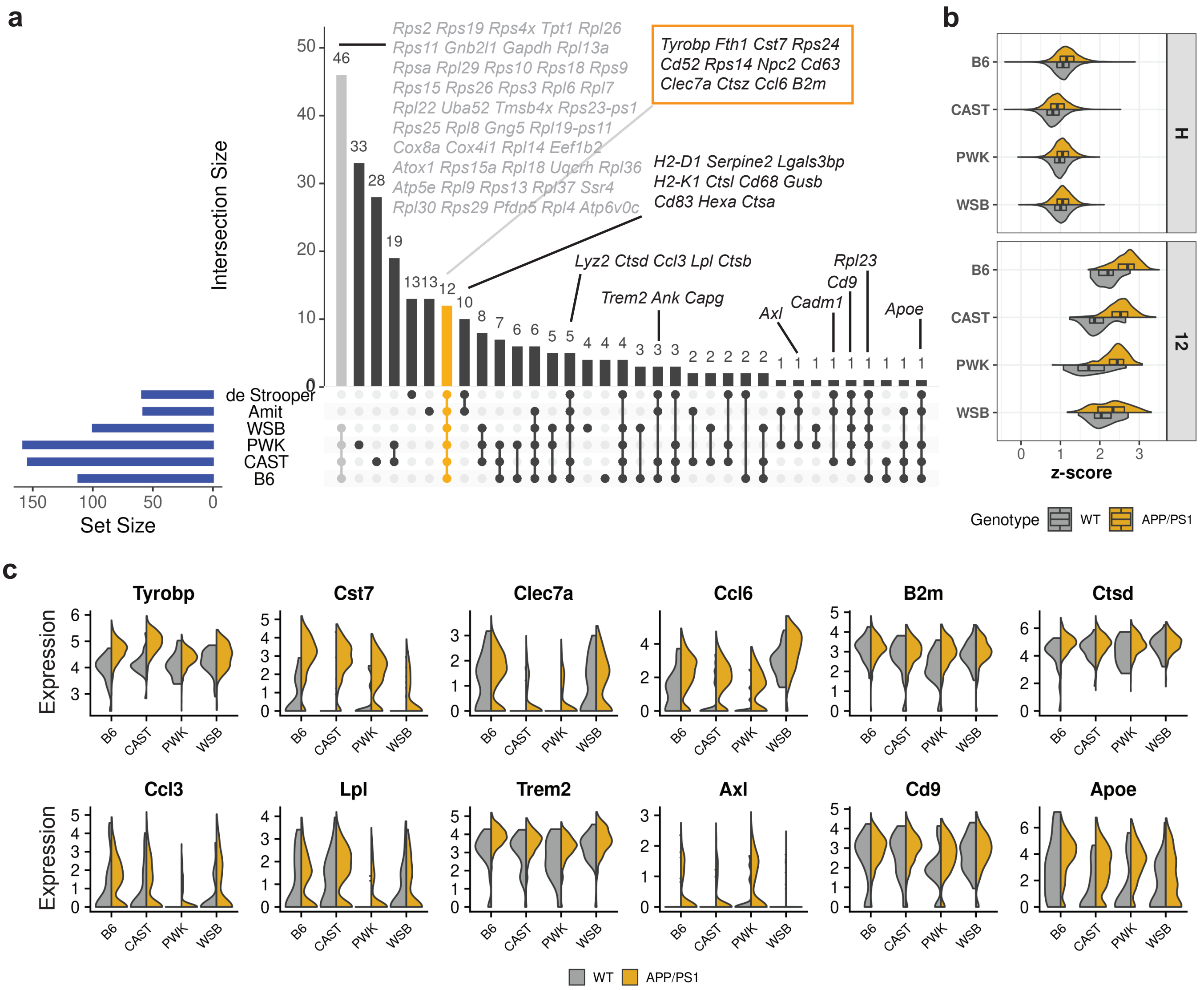

Fig. S6 | Strain-specific gene expression in disease associated microglia (cluster 12), Related to Fig.4. a, Upset plot illustrating the intersection of the top marker genes for cluster 12 in B6 and wild-derived strains integrated with top DAM marker genes from the Amit study[3] and top ARM marker genes from the de Strooper study[8]. The genes in selected intersections are detailed in the plot. Core genes shared by all datasets are highlighted in the orange box; genes shared in only between B6 and wild-derived strains from our study are colored in grey. **b**, Violin-box plots showing the enrichment z-score of core marker genes in cluster H (homeostatic) and cluster 12 for each strain and genotype. Significant strain and genotype effects were detected for each cluster ($p \approx 0$, two-way ANOVA). **c**, Violin plots showing the expression of selected marker genes in cluster 12 in each strain and genotype.

Figure S7

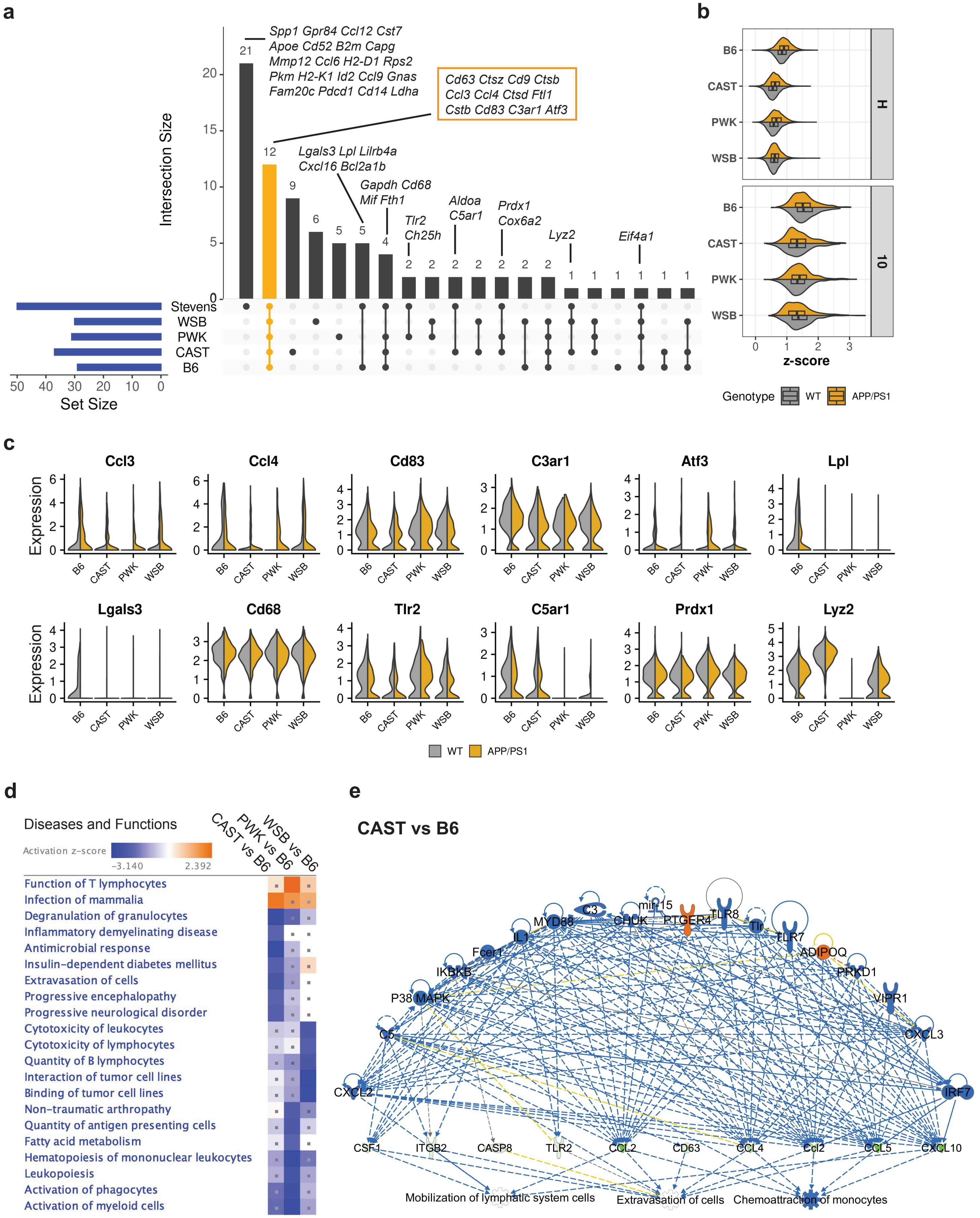

a
Spp1 Gpr84 Ccl12 Cst7
Apoe Cd52 B2m Capg
Mmp12 Ccl6 H2-D1 Rps2
Pkm H2-K1 Id2 Ccl9 Gnas
Fam20c Pdcd1 Cd14 Ldha
Cd63 Ctsz Cd9 Ctsb
Ccl3 Ccl4 Ctsd Ftl1
Cstb Cd83 C3ar1 Atf3
Lgals3 Lpl Lilrb4a
Cxcl16 Bcl2a1b
Gapdh Cd68
Mif Fth1
Tlr2
Ch25h
Aldoa
C5ar1
Prdx1
Cox6a2
Lyz2
Eif4a1
Intersection Size
Set Size
Stevens
WSB
PWK
CAST
B6
b
H
10
z-score
Genotype
WT
APP/PS1
c
Ccl3
Ccl4
Cd83
C3ar1
Atf3
Lpl
Lgals3
Cd68
Tlr2
C5ar1
Prdx1
Lyz2
Expression
WT
APP/PS1
d
Diseases and Functions
Activation z-score
-3.140
2.392
CAST vs B6
PWK vs B6
WSB vs B6
Function of T lymphocytes
Infection of mammalia
Degranulation of granulocytes
Inflammatory demyelinating disease
Antimicrobial response
Insulin-dependent diabetes mellitus
Extravasation of cells
Progressive encephalopathy
Progressive neurological disorder
Cytotoxicity of leukocytes
Cytotoxicity of lymphocytes
Quantity of B lymphocytes
Interaction of tumor cell lines
Binding of tumor cell lines
Non-traumatic arthropathy
Quantity of antigen presenting cells
Fatty acid metabolism
Hematopoiesis of mononuclear leukocytes
Leukopoiesis
Activation of phagocytes
Activation of myeloid cells
e
CAST vs B6
MYD88
C3
mir-15
CHUK
PTGER4
TLR8
Tlr
TLR7
IL1
Fcer1
ADIPOQ
IKBKB
PRKD1
P38 MAPK
VIPR1
C5
CXCL3
CXCL2
IRF7
CSF1
ITGB2
CASP8
TLR2
CCL2
CD63
CCL4
Ccl2
CCL5
CXCL10
Mobilization of lymphatic system cells
Extravasation of cells
Chemoattraction of monocytes

Fig. S7 | Strain-specific gene expression in *Ccl3*^{high}/*Ccl4*^{high} microglia (cluster 10), Related to Fig.2. a, Upset plot illustrating the intersection of the top *Ccl3*^{high}/*Ccl4*^{high} marker genes in B6 and wild-derived strains integrated with top marker genes in Aging OA2 cluster from the Stevens study[15]. The genes in selected intersections were detailed in the plot. Core genes shared by all datasets were highlighted in the orange box; genes only shared between B6 and wild-derived strains in our study were colored in grey. **b**, Violin-box plots showing the enrichment z-score of core *Ccl3*^{high}/*Ccl4*^{high} marker genes in cluster H (homeostatic) and cluster 10 for each strain and genotype. Significant strain and genotype effects were detected for each cluster ($p \approx 0$, two-way ANOVA). **c**, Violin plots showing the expression of selected marker genes for each strain and genotype. **d**, Heat map summarizing top 21 significantly enriched Diseases and Functions terms based on DE genes from comparisons of wild-derived vs B6 mice (pval-BH < 0.05, |z-score| ≥ 2). The dot indicates the enrichment of Diseases and Functions term was not significant for a given comparison (pval-BH ≥ 0.05). **e**, Selected Regulatory Effects for CAST vs B6 highlighting network of 'Mobilization of lymphatic system cells', 'Extravasation of cells' and 'Chemoattraction of monocytes'. The color code is the same as described in Fig. 3d-e.
