# Supplementary material for: Natural genetic variation determines microglia heterogeneity in wild-derived mouse models of Alzheimer’s disease
Source: Cell Rep. Author manuscript; Available in PMC 2021 Mar 7. (PMC7937391; doi:10.1016/j.celrep.2021.108739)
Supplement: 8 [file NIHMS1672405-supplement-8.pdf]

Cell Reports

Resource

Natural genetic variation determines microglia heterogeneity in wild-derived mouse models of Alzheimer's disease

Graphical Abstract

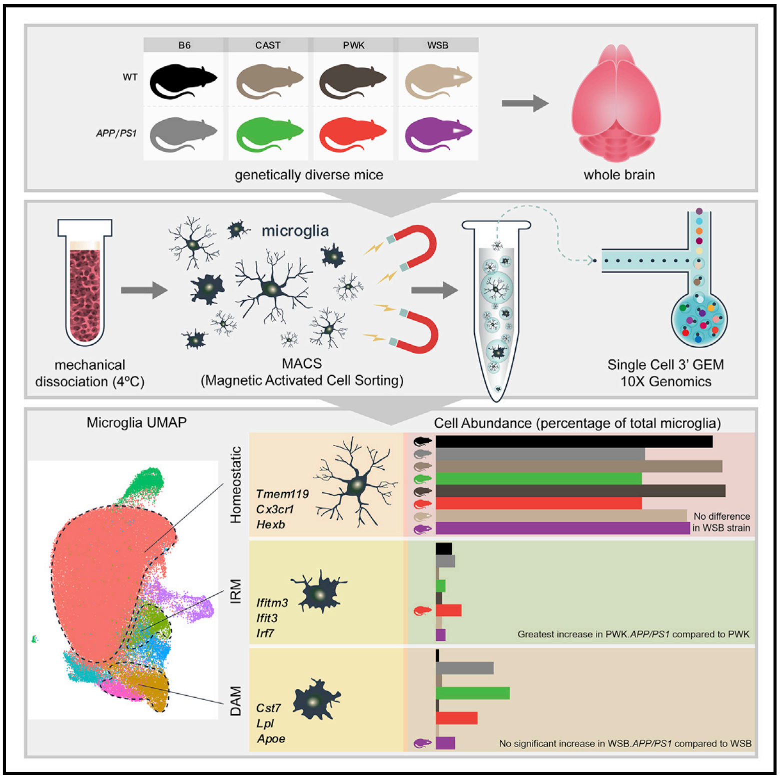

Highlights

- Genetic diversity of mouse strain is a major driver of variation in brain myeloid cells
- WSB.*APP/PS1* did not exhibit an increase in DAM or a decrease in homeostatic microglia
- PWK.*APP/PS1* is the only strain to show a significant increase in IRM compared to WT
- Core marker genes for microglia subtypes are highlighted in mouse and human studies

Authors

Hongtian Stanley Yang, Kristen D. Onos, Kwangbom Choi, Kelly J. Keezer, Daniel A. Skelly, Gregory W. Carter, Gareth R. Howell

Correspondence

gareth.howell@jax.org

In Brief

Neuroinflammation is a key component of Alzheimer's disease. Yang et al. perform single-cell sequencing of microglia in wild-derived mouse strains that carry amyloid and show that these strains differ from the commonly used strains, exhibiting significant variation in abundance of microglial subtypes, including numbers of disease-associated and interferon-responding microglia.

Yang et al., 2021, Cell Reports *34*, 108739
February 9, 2021 © 2021 The Authors.
https://doi.org/10.1016/j.celrep.2021.108739

CellPress

Cell Reports

CellPress
OPEN ACCESS

Resource

Natural genetic variation determines microglia heterogeneity in wild-derived mouse models of Alzheimer's disease

Hongtian Stanley Yang,[1,4] Kristen D. Onos,[1,4] Kwangbom Choi,[1] Kelly J. Keezer,[1] Daniel A. Skelly,[1] Gregory W. Carter,[1,2,3] and Gareth R. Howell[1,2,3,5,*]

[1]The Jackson Laboratory, Bar Harbor, ME 04609, USA
[2]Sackler School of Graduate Biomedical Sciences, Tufts University School of Medicine, Boston, MA 02111, USA
[3]Graduate School of Biomedical Sciences and Engineering, University of Maine, Orono, ME 04469, USA
[4]These authors contributed equally
[5]Lead contact
*Correspondence: gareth.howell@jax.org
https://doi.org/10.1016/j.celrep.2021.108739

SUMMARY

Genetic and genome-wide association studies suggest a central role for microglia in Alzheimer's disease (AD). However, single-cell RNA sequencing (scRNA-seq) of microglia in mice, a key preclinical model, has shown mixed results regarding translatability to human studies. To address this, scRNA-seq of microglia from C57BL/6J (B6) and wild-derived strains (WSB/EiJ, CAST/EiJ, and PWK/PhJ) with and without *APP/PS1* demonstrates that genetic diversity significantly alters features and dynamics of microglia in baseline neuroimmune functions and in response to amyloidosis. Results show significant variation in the abundance of microglial subtypes or states, including numbers of previously identified disease-associated and interferon-responding microglia, across the strains. For each subtype, significant differences in the expression of many genes are observed in wild-derived strains relative to B6, including 19 genes previously associated with human AD including *Apoe*, *Trem2*, and *Sorl1*. This resource is critical in the development of appropriately targeted therapeutics for AD and other neurological diseases.

INTRODUCTION

Alzheimer's disease (AD) is defined by the neuropathological accumulation of beta amyloid plaques, neurofibrillary tangles of tau, and widespread neuronal loss. AD is the most common cause of adult dementia and is characterized by a wide range of cognitive and behavioral deficits that severely impact quality of life and the ability to self-care. Recent work has re-focused the field on the contribution of brain glial cells to the initiation and spread of these disease-specific pathologies, specifically on the potential role of microglia as a causative cell type in driving disease development and progression. Human genome-wide association studies (GWASs) have identified more than 25 variants in or near genes uniquely expressed in microglia that are predicted to increase susceptibility for AD. In light of this complexity, the mouse represents a critical model system to dissect the role of microglia and other glia in AD.

There has been much debate regarding the alignment of mouse microglia to human microglia in terms of identity, diversity, and function. With the more widespread use of single-cell sequencing technology, a number of groups have suggested that the species difference is too great for conclusions drawn from mouse models to inform our understanding of human microglia (Friedman et al., 2018; Geirsdottir et al., 2019). Central to this argument is the discovery and description of a specific class of microglia in the mouse, disease-associated microglia (DAM) (Keren-Shaul et al., 2017). Based upon the current data, it is unclear whether the presence or absence of DAM in human AD patients is the result of differences in tissue collection, extraction of cells, genetic diversity of patients, subtypes of AD presented in donors, or even comorbidities (Friedman et al., 2018; Alsema et al., 2020). Recent work has demonstrated that single-nucleus RNA sequencing (RNA-seq) of stored human tissue fails to detect differences in microglia activation between AD and controls (Thrupp et al., 2020), further complicating direct comparisons between humans and mouse models.

The vast majority of mouse microglia gene datasets have been generated using the inbred laboratory strain, C57BL/6 (B6). Genetic complexity in the human population is expected to influence differences in, and the even presence of, microglia subtypes. However, inclusion of similar genetic diversity in mouse strains has not been explored. We have taken advantage of wild-derived mouse strains that exhibit natural genetic variation in AD risk genes (Onos et al., 2019). As the wild-derived strains CAST/EiJ (CAST), WSB/EiJ (WSB), and PWK/PhJ (PWK) were captured from the wild from different geographical regions for laboratory use, their genomes are closer to recapitulating the diversity of genetic variants that would exist in the natural world.

This is an open access article under the CC BY license (http://creativecommons.org/licenses/by/4.0/).

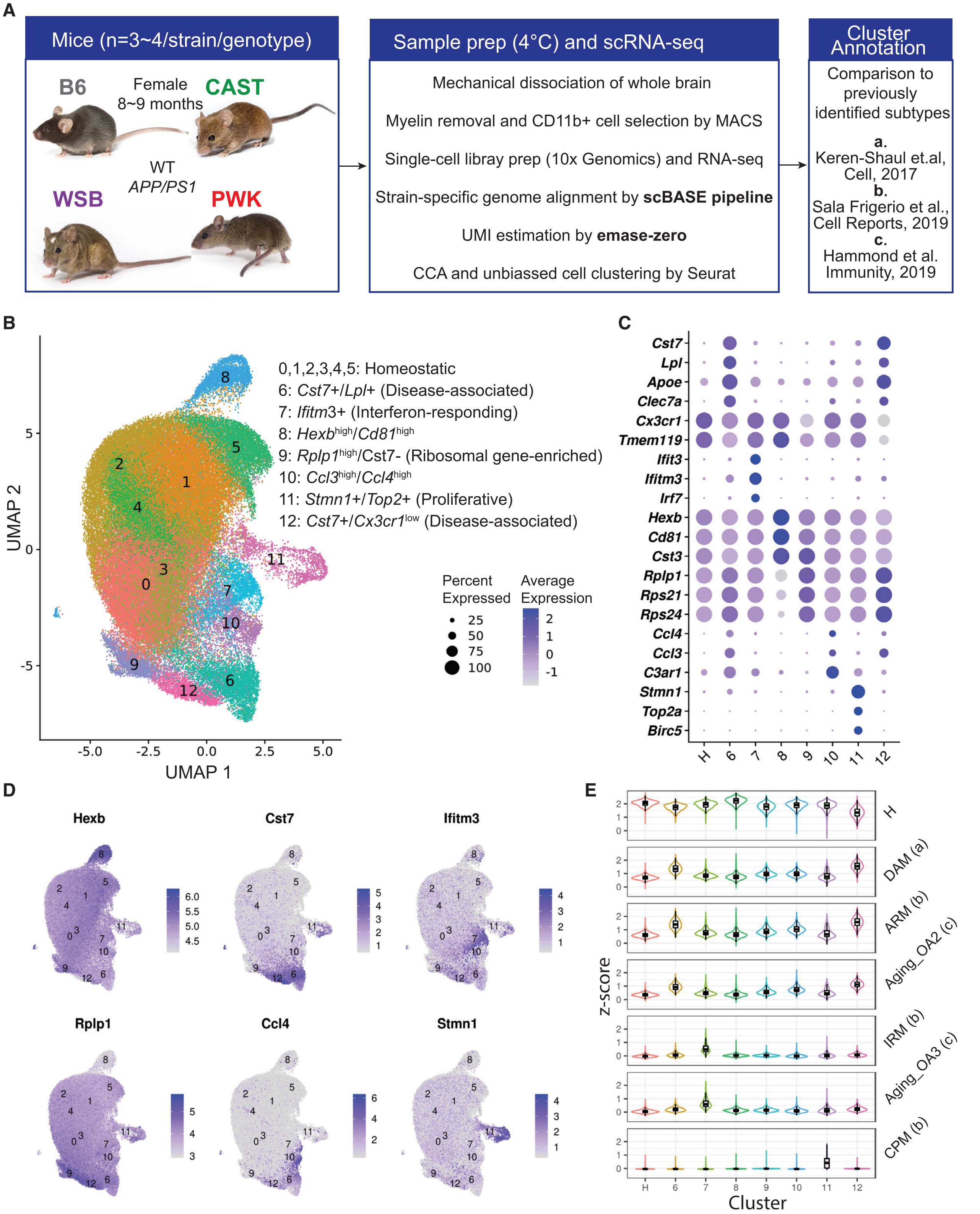

(legend on next page)

We have already demonstrated that these strains show variation in their baseline number of myeloid cells, with B6 and PWK showing twice the number of IBA1+ cells in a region of the hippocampus and cortex in comparison to CAST and WSB. Similar variation has recently been observed in B6 compared to WSB mice in the hypothalamus (Terrien et al., 2019). Therefore, it is plausible that differences in both adaptive and innate immunity may confer resilience or susceptibility to neurodegenerative diseases. The inclusion of human mutations associated with amyloid pathology, *APP*^{swe} and *PS1*^{de9} (*APP/PS1*), highlighted these strain-specific differences in neuroinflammatory responsiveness (Onos et al., 2019). For example, CAST.*APP/PS1* demonstrated a hyperproliferative phenotype with the highest density of microglia around plaques and WSB.*APP/PS1* showed the fewest plaque-associated microglia. In further support of differences in amyloid-induced microglial responses, weighted gene co-expression network analysis (WGCNA) of bulk RNA-seq data from brain hemispheres identified a microglia gene-enriched module that varied across the strains. PWK.*APP/PS1* showed the highest eigenvalues, whereas WSB.*APP/PS1* showed the lowest. These variations in both microglial phenotypes and susceptibility to neuronal cell loss suggest our wild-derived AD panel provides a unique opportunity to understand the role of microglia biology on neurodegeneration. To aid in these efforts, we have now developed a single myeloid cell data resource from wild-derived mouse strains that supports the importance of including natural genetic variation to dissect the roles of microglia model microglial biology in AD.

RESULTS

Natural genetic variation shapes the transcriptome landscape of brain myeloid cells

In order to understand microglia diversity present in wild-derived strains compared to B6, we performed single-cell RNA-seq (scRNA-seq) on brain myeloid cells isolated from female 9-month-old B6.*APP/PS1*, CAST.*APP/PS1*, PWK.*APP/PS1*, and WSB.*APP/PS1* and wild-type (WT) controls. We prioritized female mice as they showed the most variation in AD-relevant phenotypes at this age compared to males. Recent work has also suggested that sex-specific microglia differences are primarily in threshold to activation, with microglia progressing more quickly in females compared tomales (Sala Frigerio et al., 2019). Briefly, we performed mechanical dissociation (Bohlen et al., 2019) on brains to obtain a single-cell suspension for myeloid cell enrichment using magnetic-activated cell sorting (MACS) with CD11b microbeads. All steps were performed on ice or at 4°C to minimize tissue dissociation-related microglia activation (Wu et al., 2017). Single myeloid cell RNA libraries were generated using 10x Genomics v3 chemistry and sequenced by Illumina Nova-seq S2 sequencer (see method details). Fastq files were aligned to customized strain-specific genomes using scBASE pipeline (Choi et al., 2019), and gene counts were estimated by emaze-zero software (Raghupathy et al., 2018) (Figure 1A). Gene count matrix and downstream clustering analysis was processed using the Seurat package after removing low-quality cells and contaminating non-myeloid cells (Figures S1A and S1B). Overall, 91,201 myeloid cells were integrated across the strains (Figure S1C), with 87,746 identified as microglia (Figure S1D). No significant differences in yield of myeloid cells or microglia between the strains were observed (Figures S1C and S1D), despite differences in *Itgam* (Cd11b) gene expression determined from our previous bulk RNA-seq study (Onos et al., 2019) (Figure S1E) and from this study (Figure S1F). Gene expression profiles were integrated using canonical correlation analysis (CCA) (Stuart and Satija, 2019), and myeloid cells from each strain were clustered together, allowing for direct comparison of cell types between strains (Figures S2A–S2C). A total of four major myeloid cell clusters were defined—microglia (96.2%), perivascular macrophages (1.5%), monocytes (1.5%), and neutrophils (0.8%)—based upon commonly used marker genes (Keren-Shaul et al., 2017; Yang et al., 2019) including *Tmem119*, *Itgam*, *P2ry12*, *C1qa*, *Ptprc*, *Mrc1*, *Cd74*, *Itgal*, *S100a4*, and *S100a9* (Figures S2D–S2F).

Defining microglia subtypes in genetically diverse mouse strains

Given microglia were the most common myeloid cell identified, a second round of clustering was performed to more accurately define microglia subtypes or states. Thirteen microglia clusters were annotated based on relative expression levels of marker genes such as *Tmem119*, *Cx3cr1*, *Cst7*, *Clec7a*, *Apoe*, *Ifitm3*, *Hexb*, *C3ar1*, and *Stmn1* (Figures 1B–1D; Table S1). Cluster number was assigned and ordered based on overall cell abundance. Gene expression for our clusters was then compared with microglia subtypes from previous scRNA-seq studies (Keren-Shaul et al., 2017; Hammond et al., 2019; Sala Frigerio et al., 2019) (Figure 1E) by evaluating enrichment scores of marker genes from reported microglia subtype in our clusters. In our dataset, clusters 0–5 were the most abundant and were

Figure 1. Clustering and annotation of microglia subtypes in B6 and wild-derived mice

(A) Overview of the experimental strategy.

(B) UMAP plot showed 87,746 strain-integrated microglia from all 29 mice (20,732 from B6, 24,124 from CAST, 19,702 from PWK, and 23,188 from WSB), reflecting diverse microglia subtypes including homeostatic (clusters 0–5), disease-associated (clusters 6 and 12), interferon-responding (cluster 7), *Hexb*^{high}/*Cd81*^{high} (cluster 8), ribosomal gene-enriched (cluster 9), *Ccl3*^{high}/*Ccl4*^{high} (cluster 10), and proliferative microglia (cluster 11).

(C) Dot plot showing the classical marker genes for microglia subtypes with their percentage expressed (dot size) and average expression (color intensity).

(D) UMAP plots highlighting microglia subtype marker genes including *Hexb*, *Cst7*, *Ifitm3*, *Rplp1*, *Ccl4*, and *Stmn1*.

(E) Violin boxplots showing the enrichment *Z* score for each cluster (all strains combined) based on marker genes from previously identified microglia subtypes. Microglia subtypes from previous studies for comparison include homeostatic microglia (Keren-Shaul et al., 2017; Sala Frigerio et al., 2019; Hammond et al., 2019; Gosselin et al., 2017; Butovsky and Weiner, 2018), disease- associated microglia (DAM) (Keren-Shaul et al., 2017), activated-response microglia (ARM) (Sala Frigerio et al., 2019), interferon-responding microglia (IRM) (Sala Frigerio et al., 2019), aging-associated microglia (OA2, OA3) (Hammond et al., 2019), and cycling and proliferative microglia (CPM) (Sala Frigerio et al., 2019). Significant variation in enrichment score was detected across the clusters ($p < 2 \times 10^{-16}$, one-way ANOVA), which supported identification of clusters 6 and 12 as DAM/ARM and cluster 7 as IRM.

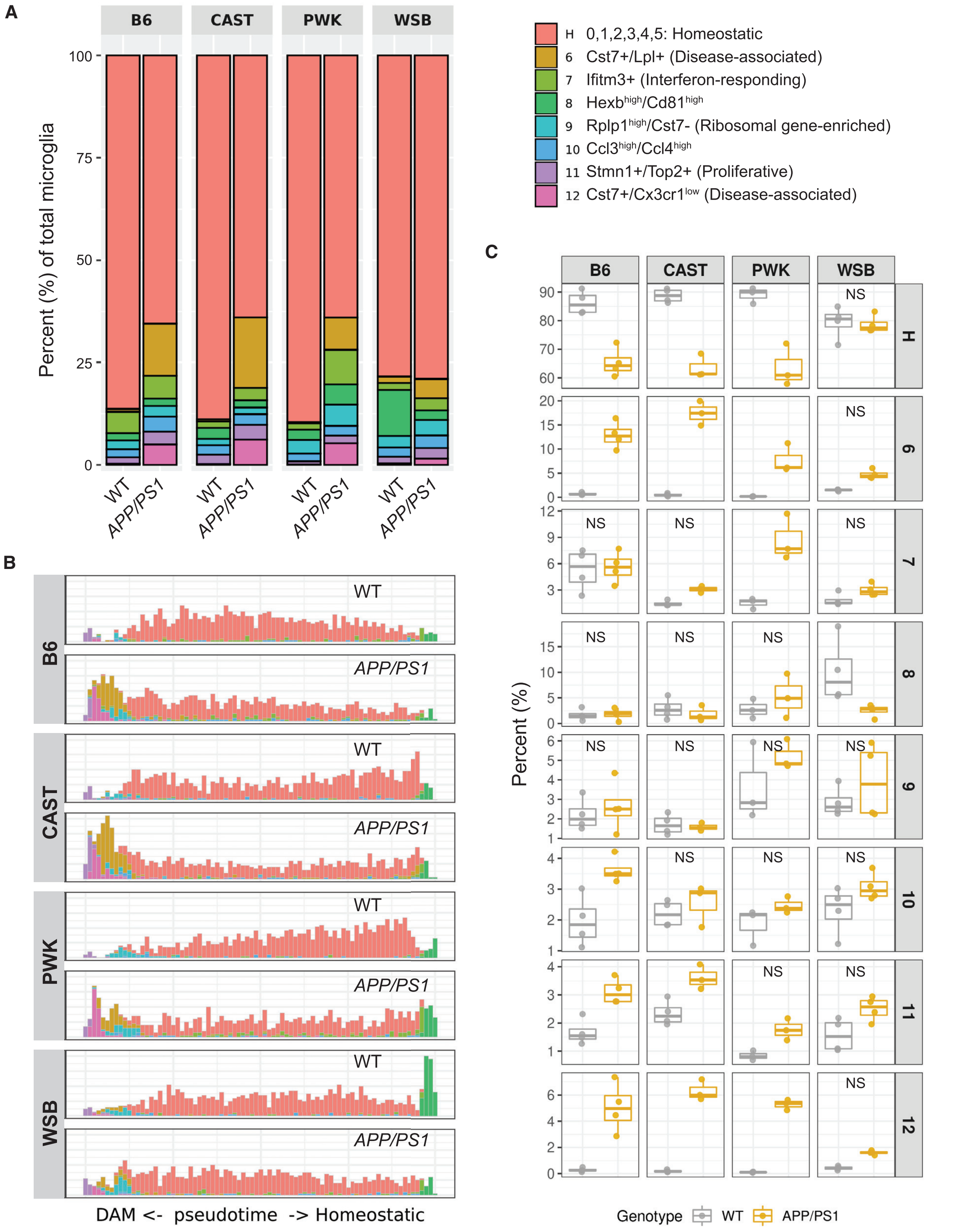

(legend on next page)

pooled (Figures S3A and S3B), collectively referred to as cluster H or homeostatic microglia. These clusters appeared consistent with "homeostatic"-like microglia, exhibiting positive overlap with 23 previously identified homeostatic marker genes (Keren-Shaul et al., 2017; Hammond et al., 2019; Sala Frigerio et al., 2019; Gosselin et al., 2017; Butovsky and Weiner, 2018) (Figure 1E). One additional cluster (cluster 8) showed similarities to cluster H, but many of the marker genes were expressed at a significantly higher level, including *Hexb*, *Cd81*, *Tmem119*, and *Cst3* (Figures 1B–1E). To our knowledge, this *Hexb*^{high}/*Cd81*^{high} cluster has not been previously identified. Clusters 6 and 12 were identified as DAM based on high expression of *Cst7*, *Lpl*, and *Clec7a* and low expression of *Cx3cr1* (Figures 1B–1D) and were more similar to previously identified DAM (Keren-Shaul et al., 2017) and activated response microglia (ARM) (Sala Frigerio et al., 2019) than any other clusters (Figure 1E). In comparison to cluster 6, cluster 12 showed lower expression of homeostatic marker genes such as *Cx3Cr1*, *Csf1r*, *Tgfbr1*, *and Tgfbr2*, as well as higher expression of *Tyrobp*, *Cst7*, and ribosomal genes (Figure S3C). The high ribosomal gene signature suggests enhanced translational activity in cluster 12 (p adj < 10^{-16}; Figure S3D). Cluster 9 also showed high expression of ribosomal genes and was in close proximity to cluster 12 in the Uniform Manifold Approximation and Projection (UMAP) plot (Figures 1B, 1C, and S3D) but did not show characteristic features of DAM. Cluster 7 was identified as interferon-responding microglia (IRM) based on marker genes that included *Ifit3*, *Ifitm3*, and *Irf7* (Figures 1B–1E) and was the cluster that showed the greatest similarity to the previously reported IRM (Sala Frigerio et al., 2019) and aging-related microglia (OA3) (Hammond et al., 2019) (Figure 1E). Cells in cluster 10, a relatively small cluster, expressed high levels of *Ccl3*, *Ccl4*, and *C3ar1* (Figures 1B–1E). Recent work identified a similar small population of microglia present during development that expand with aging or in the context of injury (Hammond et al., 2019), amyloidosis, and tauopathies (Kang et al., 2018). Cluster 11 was identified as proliferative and was enriched for *Stmn1* (Figures 1B–1E). This cluster showed the greatest similarity to the previously reported cycling and proliferating microglia (CPM) (Sala Frigerio et al., 2019) (Figure 1E). No clusters showed dramatic enrichment of immediate early genes, suggesting the MACS-based isolation method did not cause aberrant microglia activation (Wu et al., 2017) (Figure S4).

Wild-derived strains reveal transcriptomic variation in microglia subtypes

Next, we examined variation in microglia subtypes by comparing the percentage of cells in each cluster between each strain/genotype (Figures 2A, S5A, and S5B; Table S2). This was paired with trajectory inference analysis, where all eight subtypes were plotted across pseudotime to predict subtype transition (Figure 2B). The percentage of homeostatic microglia (cluster H) was significantly decreased in *APP/PS1* mice of B6, CAST, and PWK strains compared to their WT counterparts. However, this was not the case for WSB.*APP/PS1* mice, which showed a similar abundance of homeostatic microglia to WSB WT (Figure 2C).

Trajectory inference analysis predicted a transition of homeostatic microglia to the *Hexb*^{high}/*Cd81*^{high} microglia and DAM (Figure 2B). This suggests that differences in homeostatic clusters between strains may correspond to differences in transitions to other subtypes or states. There was a significantly greater percentage of *Hexb*^{high}/*Cd81*^{high} microglia (cluster 8) in WSB WT mice compared to other WT strains (Figure 2C), and these were largely absent in WSB.*APP/PS1*. Importantly, while the percentage of DAM (clusters 6 and 12) was robustly increased in *APP/PS1* mice of B6, CAST, and PWK compared to their WT counterparts, there was no significant increase in WSB.*APP/PS1* mice compared to their WT control. In addition, the percentage of IRM (cluster 7) differed between strains. PWK.*APP/PS1* mice exhibited a significantly greater proportion of IRM in comparison with PWK WT mice. This significant *APP/PS1*-dependent increase was not observed in other strains. B6.*APP/PS1* was the only strain to show a genotype-specific increase in the percentage of *Ccl3/Ccl4*-enriched cells (cluster 10). Finally, B6.*APP/PS1* and CAST.*APP/PS1* showed a significant increase in the percentage of proliferative microglia (cluster 11) compared to their WT counterparts (Figure 2C). Collectively, these analyses show that genetic diversity resulted in significant differences in the abundance of microglial subtypes in our wild-derived AD panel compared to B6.

Strain-driven transcriptome diversity predicts functional diversity of microglia subtypes

Despite the consistency of expression of marker genes within microglial clusters, initial clustering suggested widespread gene expression differences among the strains (Figure S2A). These differences could be critical for the variation we observed in amyloid-induced outcomes (Onos et al., 2019). Given their previous association to aging and AD, we chose to focus on homeostatic (cluster H), DAM (clusters 6 and 12), IRM (cluster 7), and *Ccl3/Ccl4*-enriched (cluster 10) subtypes.

We first evaluated strain and genotype differences in cluster H. To do this, we calcuated enrichment scores based on the average expression of a set of 23 homeostatic marker genes (curated from previous studies; Keren-Shaul et al., 2017; Sala Frigerio et al., 2019; Hammond et al., 2019; Gosselin et al., 2017; Butovsky and Weiner, 2018; method details). As expected, cluster H and 8 (*Hexb*^{high}/*Cd81*^{high}) were highly enriched for these homeostatic marker genes compared to cluster 6 (DAM)

Figure 2. The abundance of microglia subtypes is highly variable in B6 and wild-derived strains

(A) Percentage of microglia subtypes in WT and *APP/PS1* mice of B6, CAST, PWK, and WSB.

(B) Histogram of the pseudotime (the first dimension of the diffusion map) showing the distribution of 1,000 microglia randomly sampled from each group.

(C) Boxplots showing the percentage of microglia subtypes in all groups of mice. Strain, genotype, and strain-by-genotype effects were assessed by 2-way ANOVA followed by Tukey's post hoc test. All comparisons (comparing WT and *APP/PS1* within each strain for a given cluster) were significant (adjusted p value [p. adj] < 0.05) except for those labeled with NS (not significant, p. adj ≥ 0.05). Detailed p. adj values and confidence intervals for within and across strain/genotype comparisons are reported in Table S2.

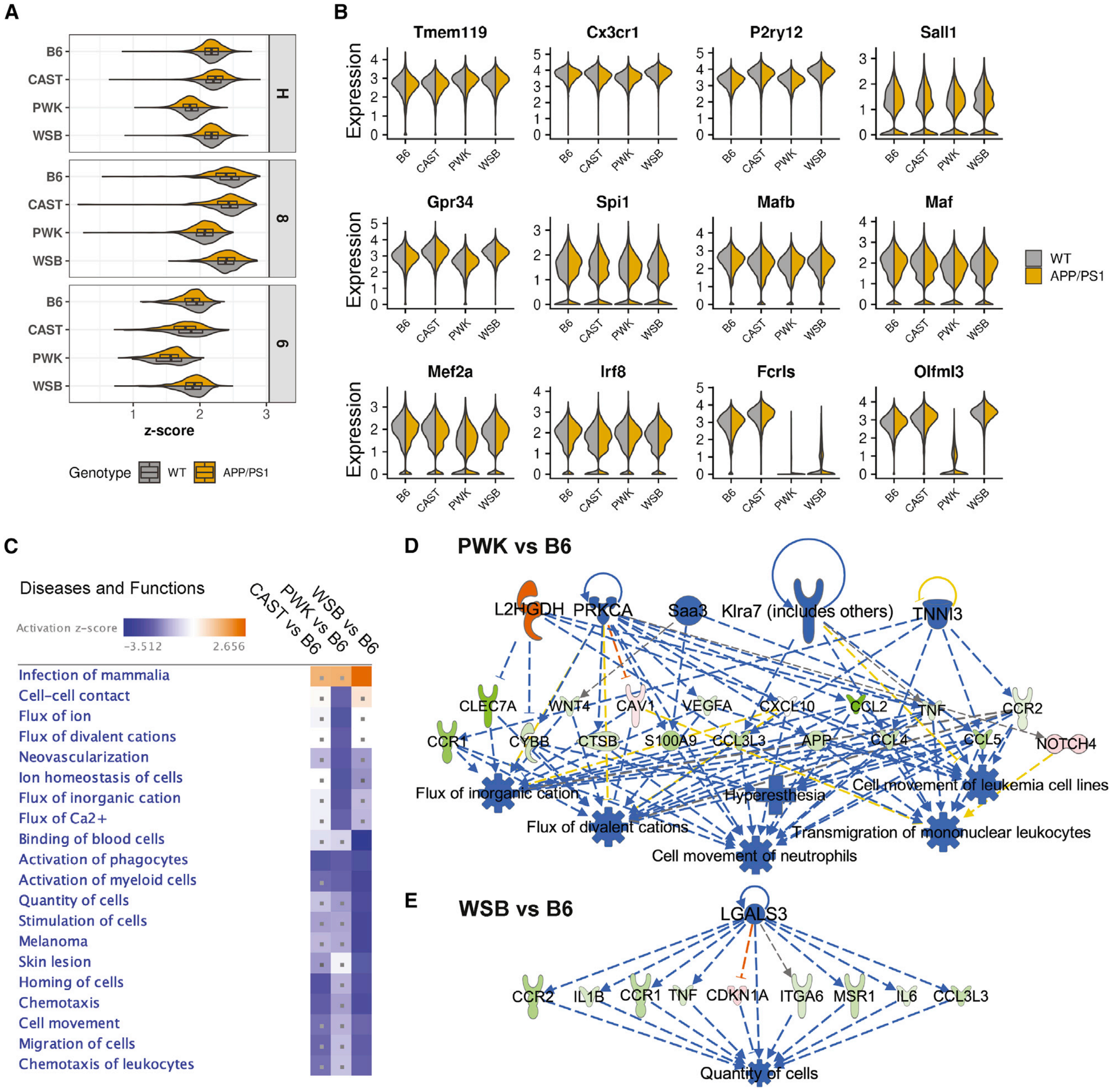

Figure 3. Strain-specific gene expression of homeostatic microglia

(A) Violin boxplots showing the enrichment *Z* score of 23 classical homeostatic microglia markers (Method details) in homeostatic (clusters 0–5), *Hexb*^{high}/*Cd81*^{high} (cluster 8), and DAM (cluster 6) in each strain and genotype. Significant strain and genotype effect was detected for each cluster ($p \approx 0$, two-way ANOVA).

(B) Violin plots showing the expression of homeostatic microglia marker genes in each strain and genotype.

(C) Heatmap summarizing top 20 significantly enriched terms of diseases and functions based on DE genes from comparisons of wild-derived versus B6 samples (corrected p value using Benjamini-Hochberg FDR (pval-BH) < 0.05, and $|Z \text{ score}| \geq 2$). The dot indicates the enrichment of diseases and functions term is not significant for a given comparison (pval-BH $\geq$ 0.05).

(D and E) Examples of REs for PWK versus B6 (D) and WSB versus B6 (E) highlighting upstream regulators (top), downstream targets (middle), and diseases and functions (bottom). The orange and blue colors indicate predicted up- or down-regulation of an upstream regulator or a diseases and functions term for a given comparison of wild-derived strain to B6. The red and green colors show up- or down-regulation of the downstream targets as DE genes comparing a given wild-derived strain to B6.

CellPress
OPEN ACCESS

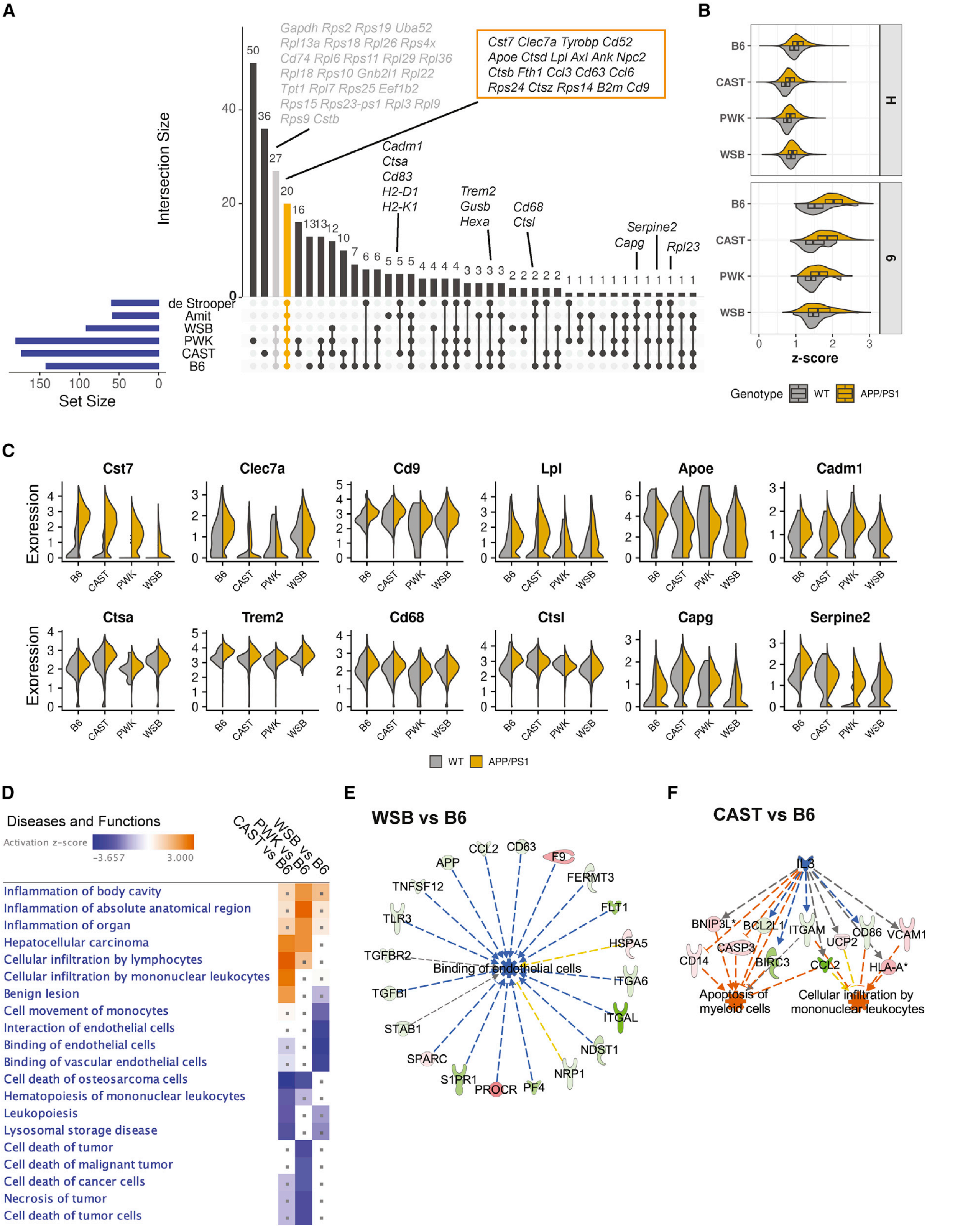

(legend on next page)

(Figure 3A). However, strain-specific expression patterns were observed in cluster H and 8. PWK showed the lowest enrichment of homeostatic marker genes in clusters H and 8 across the strains ($p \approx 0$, two-way ANOVA). Of the 23 genes, *Frls* and *Olfml3* exhibited striking strain-specific differences. *Fcrls* showed little to no expression in cluster H in PWK and WSB, while *Olfml3* showed little expression in PWK (Figure 3B). To further understand the underlying strain-specific differences in cluster H, we determined differentially expressed (DE) genes comparing cluster H gene expression between wild-derived strains and B6 (Table S3). We then performed diseases and functions analysis on the DE genes in ingenuity pathway analysis (IPA) to predict how strain-specific differences in gene expression may lead to differences in microglia function (Figure 3C). We also performed regulatory effect (RE) analysis (IPA) to predict the upstream regulator(s) that may drive such functional differences for each strain (Figures 3D and 3E). As an example, diseases and functions analysis predicted a downregulation in PWK compared to other strains in terms related to ion channels (''flux of divalent cations,'' ''flux of ion,'' ''ion homeostasis of cells,'' ''flux of inorganic cation,'' and ''flux of Ca^{2+}''; Figure 3D). This included downregulation of *Clec7a*, *Cybb*, *Wnt4*, and *Ctsb*, whose expressions are predicted to be mediated by upstream regulators *L2hgdh*, *Prkca*, *Saa3*, *Klra7*, and *Tnni3*. Homeostatic microglia are considered to be in a sensing state (Gomez-Nicola and Perry, 2015), equipped to detect environmental changes in order to respond to a variety of stimuli. At the center of this transformation is the identification of several surface channels and receptors that are critical for entry of calcium ions (Sharma and Ping, 2014; Thei et al., 2018). Thus, PWKs are predicted to be a novel strain in which to understand differences related to this process. As a second example, diseases and functions analysis predicted a downregulation in homeostatic microglia in WSB compared to the other strains centered on myeloid cell number (''quantity of cells'' and ''stimulation of cells''; Figure 3E). This includes downregulation of *Ccr2*, *Il1b*, *Tnf*, and *Il6* mediated by the upstream regulator *Lgals3*. This supports previous work that shows WSBs have fewer microglia than B6 in the specific brain regions (Onos et al., 2019).

DAM and IRM are the more prominent microglia subtypes previously implicated in aging and AD (Keren-Shaul et al., 2017; Hammond et al., 2019; Roy et al., 2020). As our study is the first to use genetically diverse mouse strains, we sought to understand the similarities and differences between these microglia subtypes with previous mouse datasets. We first compared the marker genes (Table S4) defining cluster 6 with the top marker genes from the Amit study (DAM) (Keren-Shaul et al., 2017) and the de Strooper study (ARM) (Sala Frigerio et al., 2019) (Figure 4A; Table S5). This identified a core set of 20 genes conserved across all datasets and included *Cst7*, *Clec7a*, *Tyrobp*, and *Lpl*. Genes that were present only in our dataset were primarily ribosomal and, thus, may be reflective of differences in metabolic status of the cells at the time of sample collection, the mouse models used, and/or the library generation and sequencing platforms. Interestingly, *Trem2*, a well-known marker gene in previously characterized DAM (Keren-Shaul et al., 2017) and ARM (Sala Frigerio et al., 2019), was not among the top DAM markers in cluster 6 cells from PWK and WSB.

Next, we used the 20 core DAM genes to determine how the general characteristics of DAM change across strain and genotype. Similary, we calculated enrichment scores based on the average expression of the 20 core genes in cluster 6 for WT and *APP/PS1* samples for each strain (Figure 4B). Scores were also calculated for cluster H as a control. As expected, the enrichment scores were highest for cluster 6 compared to the homeostatic cluster H, irrespective of strain or genotype. Within cluster 6, B6 showed the highest enrichment score, followed by CAST and then PWK and WSB. Within the strain, enrichment scores were higher in the presence of amyloid (*APP/PS1*) compared to WT. These data showed strain and genotype affected not only the DAM cell abundance (Figure 2C), but also the extent of ''DAM-ness.'' Further interrogation of the 20 core DAM genes highlighted strain- and genotype-specific differences in expression of individual core genes. For example, *Cst7* was highest in B6.*APP/PS1* and lowest in WSB.*APP/PS1* (Figure 4C). In contrast, B6.*APP/PS1* and WSB.*APP/PS1* strains showed the highest expression of *Clec7a*, while there was only low expression in CAST.*APP/PS1* and PWK.*APP/PS1* samples (Figure 4C). A similar analysis, comparing marker genes for cluster 12 to those in the Amit and de Strooper studies, revealed a smaller set of core genes (compared to cluster 6 analysis) (Figure S6; Table S5), further highlighting the potential that microglia in clusters 6 and 12 form distinct subtypes. Enrichment score analyses and expression of the core genes showed strain-, genotype-, and strain-by-genotype-specific patterns (Figures S6B [$p \approx 0$, two-way ANOVA] and S6C).

DE genes were determined for cluster 6 by comparing wild-derived strains to B6 (Table S3). Diseases and functions and RE analyses (IPA) were again employed to predict the functional consequences of gene expression differences (summarized in Figure 4D). WSB showed a significant downregulation of a network of genes related to ''binding of endothelial cells''

Figure 4. Strain-specific gene expression of DAM (cluster 6)

(A) Upset plot illustrating the intersection of the top DAM signature genes in B6 and wild-derived strains and top signature genes defining DAM from the Amit study (Keren-Shaul et al., 2017) and ARM in the de Strooper study (Sala Frigerio et al., 2019). Specific genes in selected intersections are detailed on the plot. Core genes shared by all datasets are highlighted in the orange box; genes shared only in our B6 and wild-derived strains are colored in gray.

(B) Violin boxplots showing the enrichment *Z* score of the core DAM genes in cluster H (homeostatic) and cluster 6 for each strain and genotype. Significant strain and genotype effects were detected ($p \approx 0$, two-way ANOVA).

(C) Violin plots showing the expression of selected DAM core genes in cluster 6 for each strain and genotype.

(D) Heatmap summarizing top 20 significantly enriched diseases and functions (IPA) terms based on DE genes from comparisons of wild-derived versus B6 mice (pval-BH < 0.05, $|Z \text{ score}| \geq 2$). The dot indicates the enrichment of diseases and functions term is not significant for a given comparison (pval-BH $\geq$ 0.05).

(E) Example of an RE for WSB versus B6 highlighting the of ''binding of endothelial cells.''

(F) Example of an RE for CAST versus B6 highlighting network of ''apoptosis of myeloid cells'' and ''cellular infiltration by mononuclear leukocytes.'' The color code is the same as described in Figures 3D and 3E.

CellPress
OPEN ACCESS

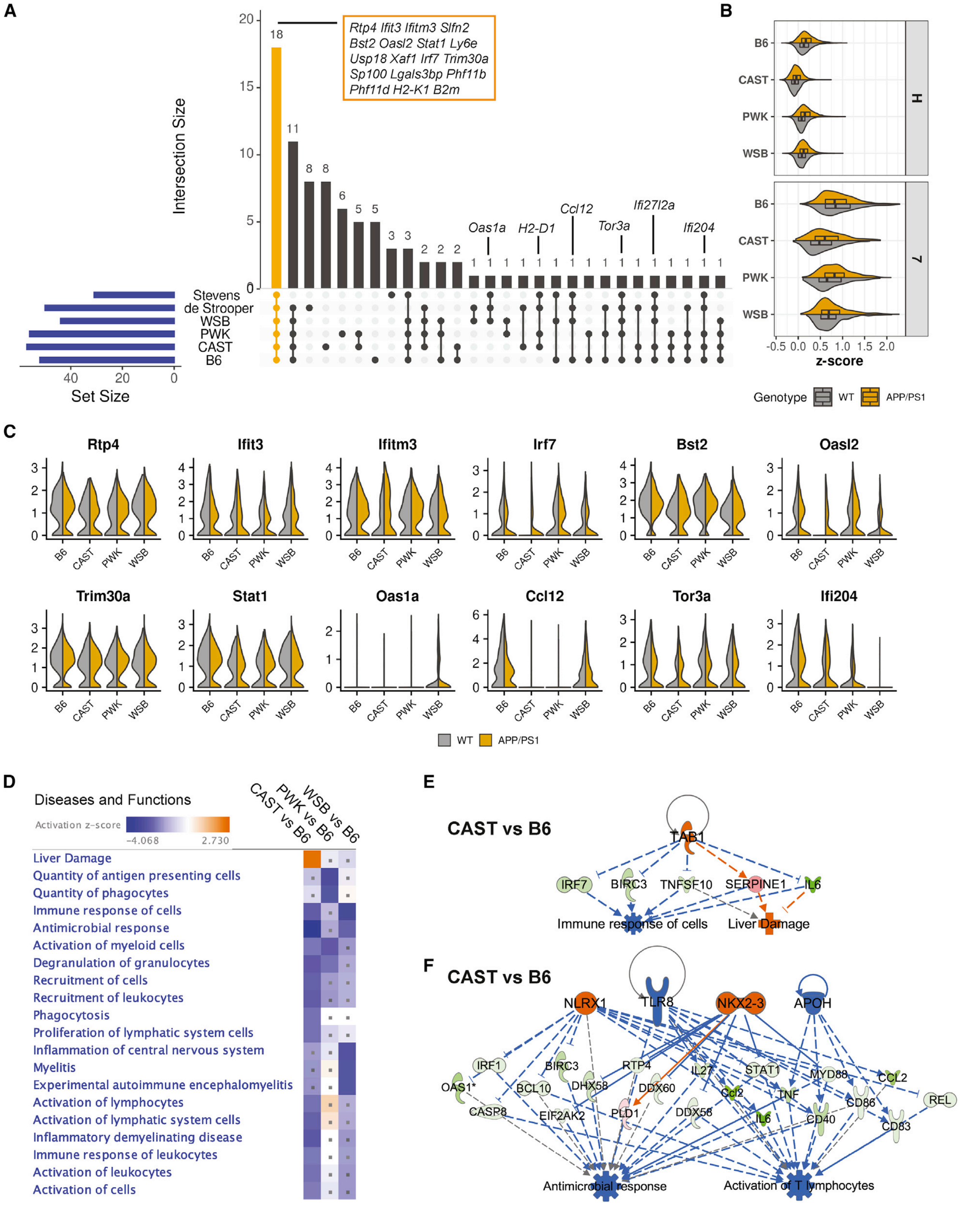

(legend on next page)

including integrins (e.g., *Itga6* and *Itgal*) that are necessary for the binding of myeloid cells to endothelial cells (Figure 4E). In our previous study, WSB.*APP/PS1* showed the highest levels of cerebral amyloid angiopathy (CAA), associated with vascular leakage and neuronal loss (Onos et al., 2019). Therefore, these data suggest that WSB.*APP/PS1* is an important model to understand the interplay between microglial function and vascular damage in AD. In a second example, CAST showed a significant activation of genes related to "cellular infiltration of mononuclear leukocytes," regulated by IL3 (Figure 4F). IL3 is a growth factor and cytokine involved in homing microglia to plaques and is thought to be neuroprotective (Zambrano et al., 2007). IL3 enrichment is driven by the upregulation of genes including *Vcam1*, *Cd14*, and *Casp3* in CAST DAM compared to B6 DAM.

Recent evidence supports an important role of IRM in AD and other brain disorders (Sala Frigerio et al., 2019; Hammond et al., 2019; Roy et al., 2020; Hur et al., 2020). Therefore, similar analyses to that described above for DAM were performed, comparing cluster 7 to IRM-like populations identified in the de Strooper (Sala Frigerio et al., 2019) and Stevens (Hammond et al., 2019) studies (Figure 5A; Table S5). A set of 18 core genes was identified in all IRM-like subtypes and included *Ifitm3*, *Ifit3*, and *Irf7*. Enrichment scores of these 18 genes were generally higher in *APP/PS1* compared to WT samples across the strains (Figure 5B), although significant strain-, genotype- and strain-by-genotype differences in gene expression were observed (Figure 5C). Diseases and functions analysis of DE genes comparing IRM from wild-derived strains to B6 identified multiple terms predicted to alter function (Figure 5D). For instance, a network related to "liver damage" was upregulated in CAST compared to B6 (Figure 5E). Genes in this network included *Irf7*, *Birc3*, *Tnfsf10*, *Il6*, *Serpine1*, and *Tab1* (predicted to be the upstream regulator), which have been shown to be DE in brains of AD patients (Agora Consortium, 2020) and identified as targets for therapeutics (Costa et al., 2017; Romagnoli et al., 2020; Calandria et al., 2015; Riphagen et al., 2020; Cantarella et al., 2015; Kutz et al., 2012; Caraci et al., 2012). Based on our data, CAST would be a more appropriate strain than B6 to assess drugs that target genes in this network. In contrast, "activation of lymphocytes" and "antimicrobial response" were downregulated in CAST compared to B6 (Figure 5F). Interferons are a group of cytokines secreted in response to stress or viral infection and are associated with autoimmune diseases. Patients with HIV-induced dementia exhibit increases in interferon activation (Gray et al., 1996), and the viral theory of AD has recently made a resurgence (Itzhaki et al., 2020). Upstream regulators *NLRX1*, *NKX2-2*, and *TLR8* are all related to type-1-interferon-triggering components such as *STAT1* and *MYD88*. Nucleic acid (NA)-containing amyloid fibrils can potently induce this cascade (Roy et al., 2020). Furthermore, increases in NLRX1, cytoplasmic NOD-like receptors localized to the outer membrane of mitochondria, have been associated with increased production of reactive oxygen species (Abdul-Sater et al., 2010). This suggests that strategies that compare CAST.*APP/PS1* (low expressers) with PWK.*APP/PS1* (high expressers) would be appropriate to parcel this relationship between viral immune pathways and AD.

The final cluster we focused on was cluster 10, termed *Ccl3*^{high}/*Ccl4*^{high} microglia. We compared the top marker genes for cluster 10 with an "age-related" subpopulation identified in the Stevens study (Hammond et al., 2019) (Figure S7A; Table S5). Although 12 top marker genes were common between our study and the Stevens study, 21 marker genes were unique to the Stevens study, including genes commonly associated as DAM genes (e.g., *Spp1*, *Cst7*, *Apoe*, *B2m*, and *Ccl6*). This may be due to the age difference between the mice sampled in our study (9 months old) and those sampled in the Stevens study (18 months old) and suggests this age-related *Ccl3*^{high}/*Ccl4*^{high} subpopulation polarizes toward DAM-like during aging. Enrichment analysis revealed subtle yet significant strain-, genotype- and strain-by-genotype differences (Figure S7B). Interestingly, *Lpl* expression was only present in the B6 strain (Figure S7C). Further analysis of diseases and functions found that in comparison with B6, CAST show a downregulation in pathways relevant to "multiple sclerosis," "inflammatory demyelinating disease," and "extravasation of cells" (Figures S7D and S7E). A previous study has localized these cells to the center of active demyelinating lesions in multiple sclerosis patients (Hammond et al., 2019), and they are suggested to signal to peripheral immune cells. The downregulation of this gene network in CAST mice suggests that the loss of neurons we have previously reported in CAST.*APP/PS1* (Onos et al., 2019) may be independent of damage caused by infiltrating immune cells.

Comparison of genetically diverse mouse microglia with human microglia

A critical and active area of investigation is the comparison between human and mouse microglia. Mouse models will likely play a major role in identifying potential microglia-based therapies to treat AD. Here, we chose four studies (Zhou et al., 2020; Johnson et al., 2020; Mathys et al., 2019; Olah et al., 2018) that surveyed human microglia to compare to our

Figure 5. Strain-specific gene expression of IRM

(A) Upset plot illustrating the intersection of the top IRM marker genes in B6 and wild-derived strains integrated with Aging_OA3 cluster from the Stevens study (Hammond et al., 2019) and IRM from the de Strooper study (Sala Frigerio et al., 2019). The genes in selected intersections are detailed in the plot. Core genes shared by all datasets are highlighted in the orange box.

(B) Violin boxplots showing the enrichment *Z* score of the 18 core IRM signature genes in cluster H (homeostatic) and cluster 7 (IRM) for each strain and genotype. Significant strain and genotype effects were detected for each cluster ($p \approx 0$, two-way ANOVA).

(C) Violin plots showing the expression of selected IRM core genes in cluster 7 for each strain and genotype.

(D) Heatmap summarizing top 20 significantly enriched terms of diseases and functions based on DE genes from comparisons of wild-derived versus B6 mice (pval-BH < 0.05, $|Z \text{ score}| \geq 2$). The dot indicates the enrichment of diseases and functions term was not significant for a given comparison (pval-BH $\geq$ 0.05).

(E) Example of an RE for CAST versus B6 highlighting network of "immune response of cells" and "liver damage."

(F) Example of a second RE for CAST versus B6 highlighting "antimicrobial response" and "activation of T lymphocytes." The color code is the same as described in Figures 3D and 3E.

CellPress
OPEN ACCESS

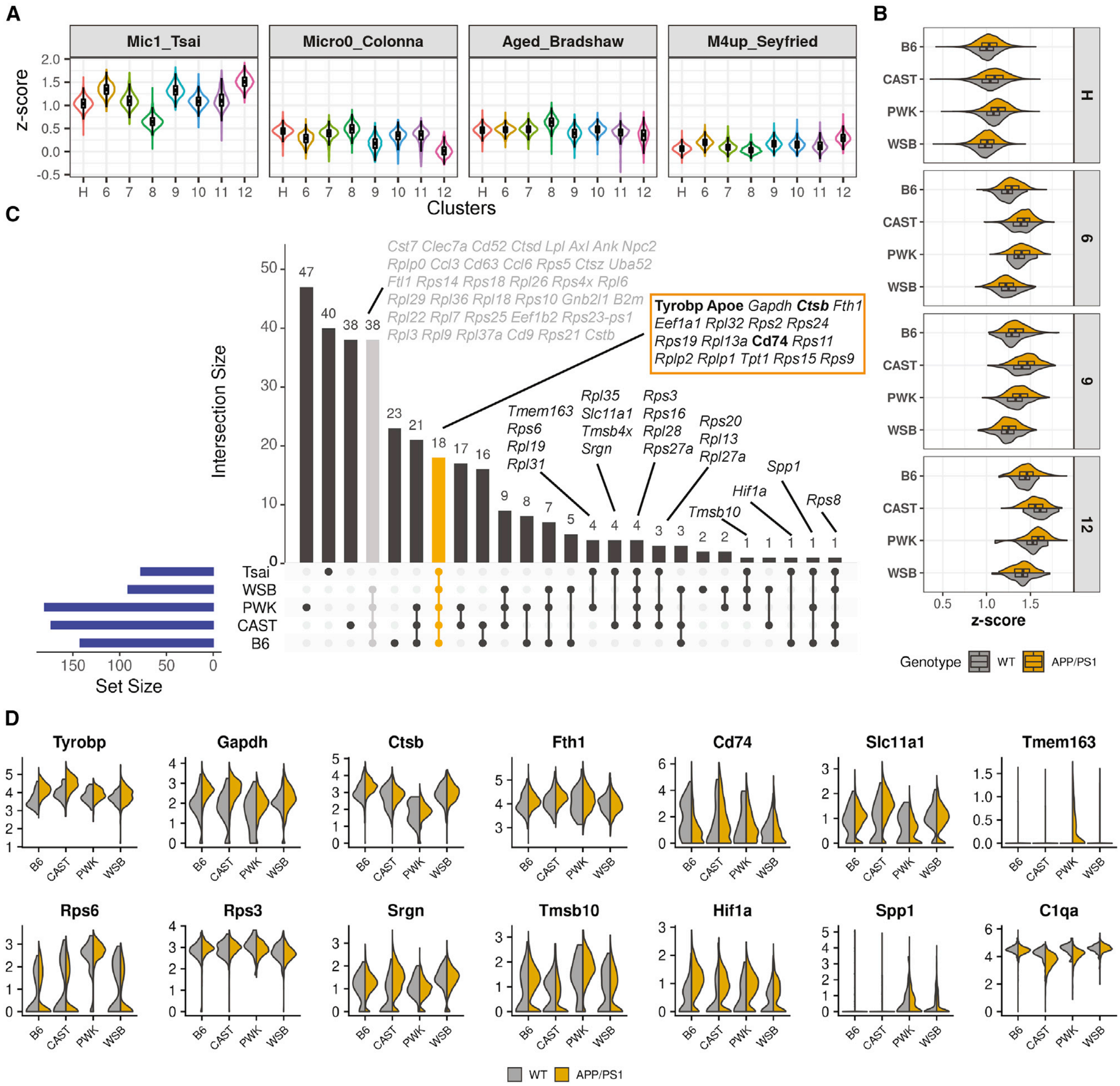

Figure 6. Comparison of cluster 6 (DAM) with human microglia

(A) Violin boxplots showing the enrichment *Z* score of the top marker genes defining microglia from four studies: Mic1 from Tsai (Mathys et al., 2019); Micro0 from Colonna (Zhou et al., 2020); aged microglia from Bradshaw (Olah et al., 2018); and M4 microglia module from Seyfried (Johnson et al., 2020).

(B) Violin boxplots showing the enrichment *Z* score of the top marker genes of the Mic1 cluster from the Tsai study for each strain and genotype. Significant strain and genotype effects were detected for each cluster ($p \approx 0$, two-way ANOVA).

(C) Upset plot illustrating the intersection of the top DAM marker genes in B6 and wild-derived strains integrated with top marker genes associated with human Mic1 cluster (Tsai) (Mathys et al., 2019). The genes in selected intersections are detailed in the plot. Core genes shared by all datasets are highlighted in the orange box; genes shared in our B6 and wild-derived strains but not Mic1 are colored in gray.

(D) Violin plots showing the expression of selected genes for each strain and genotype.

genetically diverse mouse microglia dataset. Two datasets were generated via single-nucleus RNA-seq (Mic1 from Tsai study [Mathys et al., 2019] and Micro0 from the Colonna study [Zhou et al., 2020]), one was generated via bulk RNA-seq of isolated aged microglia by Bradshaw and colleagues (Olah et al., 2018), and one (module 4, enriched for microglial genes) was obtained from the proteomics study performed by Seyfried and colleagues (Johnson et al., 2020). The marker gene set for Mic1 (Tsai) showed the highest enrichment values compared to the other three datasets, with clusters 6, 9, and 12 showing the

greatest alignment (Figure 6A). The poor alignment with Micro0 (Colonna) may be because the AD patients were enriched from common or rare variants in *TREM2*. The poor alignment with the Bradshaw and Seyfried datasets may be due to differences in sample type, preparation, and sequencing technologies.

Given the greatest similarity across the human datasets was observed from the Tsai study (Mathys et al., 2019), enrichment scores for our clusters were calculated using the Mic1 marker genes (method details). The resulting enrichment score was significantly affected by strain-, genotype-, and strain-by-genotype (Figure 6B; $p \approx 0$, two-way ANOVA). For instance, CAST and PWK displayed slightly yet significantly higher enrichment scores than B6 and WSB for clusters 6, 9, and 12 (Figure 6B). Comparisons between Mic1 and cluster 6 across all strains identified 18 core signature genes that included the classical DAM genes such as *Apoe* and *Tyrobp*, but also the ribosomal genes (Figures 6C and 6D; Table S5) that had been absent in mouse DAM datasets from previous studies (Keren-Shaul et al., 2017; Sala Frigerio et al., 2019) (Figure 4A). Interestingly, 19 of the 77 Mic1 marker genes were identified in only one or some, but not all, of the mouse strains. For instance, ribosomal proteins *Rps3*, *Rps16*, *Rpl26*, and *Rpl27a* were common between Mic1 and all wild-derived strains (CAST, WSB, and PWK) but not B6. *Tmem163* was common between Mic1 and PWK. *Spp1* was common among Mic1, PWK, and B6. These data support the use of genetically diverse mouse strains to improve the alignment to human studies. However, the Mic1 dataset lacks other classic mouse DAM genes—including *Cst7*, *Clec7a*, and *Lpl* (Figure 6C)—that have been identified in multiple mouse studies including our own (Figure 4A), suggesting further work is required to elucidate apparent species-specific differences in microglial subtypes such as DAM.

Human AD-relevant GWAS genes are differentially expressed in microglia subtypes

Variation in microglia-relevant genes is differentially associated with AD risk. However, previous studies to determine roles of GWAS genes in AD have primarily been limited to B6. Therefore, we aimed to determine whether our wild-derived AD panel provided an enhanced platform to study human AD-relevant genes using a panel of 54 GWAS genes identified in two recent meta-analyses (Table S6) (Jansen et al., 2019; De Rojas et al., 2020). A total of 36 microglia-relevant genes were detectable across our panel. Nineteen of the 36 genes (52%) were DE (false discovery rate [FDR] < 0.05) in at least one cluster comparing wild-derived strains to B6 (Figure 7A). Genes could be DE in only one cluster of one strain (e.g., *Adam10* in cluster 8, CAST versus B6; *Bin1* in cluster 8, PWK versus B6; *Inpp5d* in cluster 6, PWK versus B6; and *Pilra* in cluster 6, WSB versus B6), while other genes were DE in multiple clusters within a specific strain (e.g., *Ptk2b* and *Ndufa1* in CAST; *App* and *Sorl1* in PWK). *Scimp* and *Apoe* were DE in at least one cluster in all wild-derived strains compared with B6. The expression in WT and *APP/PS1* mice across the four strains was then determined for cluster H, DAM, and IRM (Figure 7B). This further highlighted strain- and genotype-specific differences in GWAS genes. For instance, *Sorl1* was expressed in many more cells in IRM (cluster 7) from PWK mice compared to B6, CAST, and WSB. Moreover, the relative expression level of *Sorl1* was significantly increased in PWK.*APP/PS1* compared to PWK mice. Therefore, these data further support the use of specific or contrasting wild-derived strains for more extensive and informative functional studies of AD-relevant GWAS genes.

DISCUSSION

Single-cell sequencing of microglia from wild-derived and B6 mouse strains revealed that natural genetic variation led to significant differences in populations of microglia subtype and gene expression profiles that are predicted to impact microglia biology, likely resulting in inherently different neuroimmune environments in healthy and diseased states. As with all genomic studies, these predicted differences will need to be validated and the functional consequences determined. These observed variations in microglia subtypes or states between strains likely influence, or are influenced by, other cell types including astrocytes, endothelial cells, and neurons. While these data provide further evidence for the value of mouse genetic diversity to unravel the complexity of neuroinflammation in AD, future work will need to assess additional cell types. Further, in this study, microglia from female mice at one age (9 months) were profiled. In addition to all the strain- and strain-by-genotype-specific changes observed in this dataset, sex-, brain-region- and/or age-specific changes are still to be determined.

Differences across wild-derived strains in microglia subtypes often showed downregulation of specific biological pathways in comparison to B6. While B6 has been used across biomedical research for practical and historical reasons, such work may be inherently biased to neuroimmune responses driven by a singular genetic context, with limited translation to humans. For example, B6 (as well as other commonly used strains such as DBA/2) carries a mutation in the *P2rx7* locus that severely impairs important functions of this receptor. This is thought to influence critical steps relating to induction of apoptosis and cytokine secretion. In contrast, wild-derived strains carry the "natural" variant (Adriouch et al., 2002). Another key consideration is that previous microglia sequencing projects have used the 5XFAD model. There are two versions of this model: one congenic on B6 (JR# 34848) and the other more commonly used B6.SJL mixed genetic background (JR# 34840). SJL mice carry the *Trem2*^{S148E} mutation, which means that in this 5XFAD strain, *Trem2* could be heterozygous, homozygous, or WT, influencing microglia function differently within the same study or across studies. These inconsistencies in both amyloid drivers and genetic context have likely contributed to the lack of alignment between mouse and human studies when not taken into account. Our study supports incorporating genetic diversity in this specific way to elucidate the roles of microglia in AD in conjunction with more late-onset AD-relevant variants.

We detected significant strain-, genotype- and strain-by-genotype differences. These were in both the abundance of microglia subtypes and gene expression that, in combination with pathway analysis and neuropathology, are predictive of functional differences that may be beneficial or damaging, depending on the stage of disease. For example, homeostatic microglia are typically defined as being in a sensing state, sampling the brain

CellPress
OPEN ACCESS

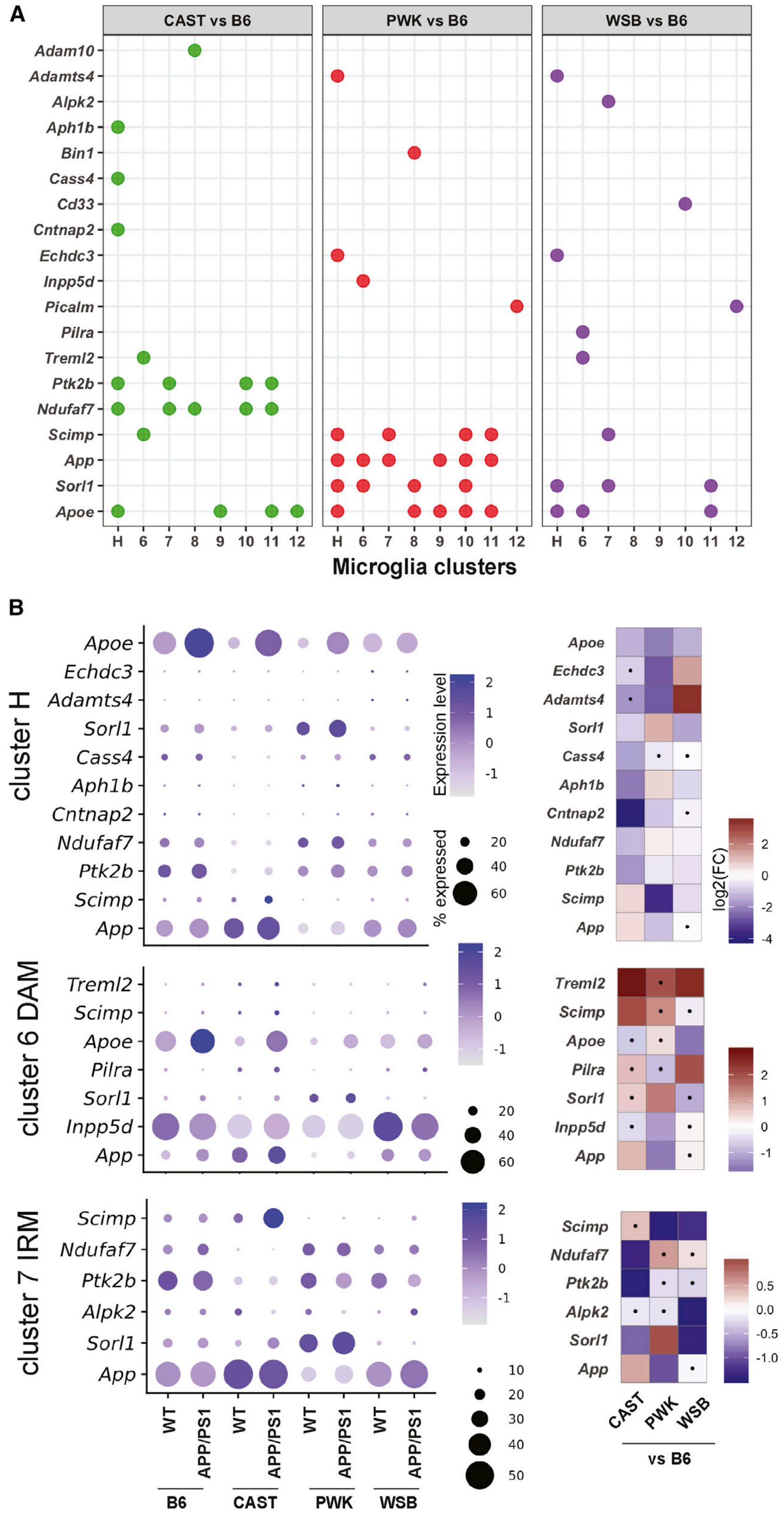

Figure 7. Microglia subtypes from wild-derived strains show differentially expressed AD-relevant GWAS genes

(A) Nineteen AD-relevant GWAS genes were DE comparing wild-derived strains to B6 for all eight microglia clusters. The dot indicates the gene was significantly DE (FDR < 0.05) comparing CAST, PWK, or WSB to B6 in a given cluster.

(B) Dot plot (left) showing the percentage of cells expressed and the expression levels of significant strain-specific DE genes in homeostatic microglia (cluster H), DAM (cluster 6), and IRM (cluster 7) across all groups. Heatmap (right) highlighting the log2-based fold change (log2FC) of the corresponding gene expression comparing CAST, PWK, and WSB to B6 in clusters H, 6, and 7. The dot in the heatmap indicates the fold change for a given comparison was not significant (FDR ≥ 0.05).

environment for debris and potential pathogens. If a signal is encountered, they quickly become "activated"—sometimes refered to as a responding state—to deal with the threat. Upon resolution, microglia are expected to revert to their surveillance role. One theory regarding the influence of microglia to disease susceptibility is that once triggered, these microglia cannot revert, becoming chronically “activated,” signaling to other local immune cells, and potentially causing damage to healthy tissue (McQuade and Blurton-Jones, 2019). Our data predict natural genetic variation influences the baseline responsiveness, efficiency of response, and reversion to surveillance. Initial clustering of microglia identified six groups of homeostatic-like microglia that were collapsed into one cluster based upon similarity of marker gene expression. However, initial clustering predicted subtle but distinct functional differences that remain to be resolved. The function of *Hexb*^{high}/*Cd81*^{high} cells (cluster 8) is not clear. Cells showed higher expression levels of *Cst3*, *Cd81*, and *Hexb* compared to the homeostatic cluster. Two small *Hexb*-related clusters that display a signature of lipid metabolism and phagocytosis have been previously reported (Keren-Shaul et al., 2017); however, those clusters do not fully align with cluster 8. Alternatively, given that pseudotime analysis suggested that this subtype transitioned in the opposite direction to activated subtypes like DAM and IRM, cells in cluster 8 may represent a microglia “reserve” pool. Interestingly, *Hexb*^{high}/*Cd81*^{high} microglia also have the highest expressions of *P2ry12*, and P2ry12-mediated chemotaxis is critical for closure of the blood-brain barrier after injury (Lou et al., 2016). In this process, homeostatic microglia elevate the expression of P2ry12 rather than transition into an activation state (such as DAM). The higher levels of this population in WSB could play a larger role in age-related health of their vasculature, and in the context of amyloid, this population may disappear as they attempt to transition to other microglia states. Overall, WSB appears to be an important genetic context to discover more about this novel *Hexb*^{high}/*Cd81*^{high} microglia subtype.

Two DAM-like clusters (clusters 6 and 12) were identified based on lower expression of *Cx3cr1*, higher expression of *Tyrobp* and *Cst7*, and increased ribosomal gene expression in cluster 12 compared to cluster 6. Two previous studies have reported two subtypes of DAM. In one study, two DAM subtypes were suggested to represent *Trem2*-specific transition states (Keren-Shaul et al., 2017), while the second study predicted proinflammatory and anti-inflammatory subtypes (Rangaraju et al., 2018). However, these differences in DAM-like cell populations were not seen in our study. This may be due to multiple reasons including sample collection and analysis methods but may also be due to the amyloid-driving transgenes used. Microglia activation and amyloid accumulation have been identified as early as 6 weeks in 5xFAD mice (Oakley et al., 2006; Onos et al., 2019; Boza-Serrano et al., 2018) but are not apparent until 4–5 months in B6.*APP/PS1* mice (Jackson et al., 2013; Chintapaludi et al., 2020). DAM populations in our wild-derived and B6 AD panel were also significantly smaller than has been previously reported in another amyloid strain, B6.*APP*^{swe}/*PS1*^{L166P} (Sierksma et al., 2020), which is also an aggressive amyloid strain with plaque accumulation observed as early as 6 weeks (Radde et al., 2006)

CAST.*APP/PS1* showed the greatest proportion of DAM (clusters 6 and 12), which is consistent with our previous work that showed *CAST.APP/PS1* had the greatest number of plaque-associated microglia (Onos et al., 2019). Given that CAST.*APP/PS1* showed significant neuronal loss in the hippocampus, this indicates a connection between a strong DAM response and neurodegeneration. However, whether the DAM response is beneficial or damaging is still to be elucidated. Interestingly, WSB.*APP/PS1*, which did not show a significant increase in DAM compared to their WT counterparts, also showed neuronal cell loss (Onos et al., 2019). Gene expression analyses predicted a downregulation of genes related to cellular interactions with endothelial cells in WSB compared to B6. CAA and vascular dysfunction were previously identified in WSB.*APP/PS1* mice (Onos et al., 2019), and CAA is thought to be independent of neuroinflammation in human AD patients (Greenberg et al., 2020). Recent work used the CSF1R inhibitor PLX5622 to deplete microglia in 5XFAD, resulting in an almost-complete loss of amyloid in the parenchyma and significant CAA and vascular leakage (Spangenberg et al., 2019). Given the presence of other microglial subtypes in WSB.*APP/PS1* mice, these data suggest DAM may be specific determinants of the balance between parenchymal- and vascular-based amyloid. In light of these findings, WSB.*APP/PS1* may be an ideal strain to dissect the relationship between amyloidosis, CAA, and vascular dysfunction in AD without the need to deplete brains of all microglia. If these differences in DAM also translate to humans, there are likely patients who show an elevated DAM response and patients who do not. This could partially explain the controversy over the alignment of DAM populations in humans and mice.

Our study highlights the importance of broadening interest in microglia subtypes beyond DAM. IRM were significantly different between strains, with only PWK.*APP/PS1* showing a significant increase compared to WT. The interferon response is a complex process that can trigger the expression of thousands of interferon-stimulated genes (ISGs). Commonly, the interferon response is thought to be triggered in response to a viral infection, and strain differences in viral response have been identified. CAST is uniquely susceptible to infections such as influenza H3N2 and monkeypox virus. In the case of influenza H3N2, despite high viral load in the lungs, CAST exhibited an abnormal response in leukocyte recruitment (Leist et al., 2016). Even at low inoculums of monkeypox virus, CAST showed rapid spread to all internal organs. This was shown to be directly related to deficiency in gamma interferon (Earl et al., 2012). In AD, the interferon response can be triggered by NA-containing plaques. In our study and other studies, IRM are defined by the presence of interferon regulator gene *Irf7* as well as ISGs *Ifitm3* and *Ifit3*. In one recent study, brain samples showed the presence of IFITM3+ microglia in NA^{+} plaques (Roy et al., 2020). Enhancing the interferon response in a B6.5xFAD exacerbated synapse loss. In contrast, our study supports a beneficial role for IFITM3+ IRM in AD: PWK.*APP/PS1* showed increased level of IRM compared to the other strains and are resilient to neurodegeneration at 8 months (Onos et al., 2019). In support of this, mice deficient for IFITM3 are more susceptible to viral infection (Kenney et al., 2019). A recent study also showed the IFITM3 modulates gamma-secretase activity in AD (Hur et al., 2020). Given

the multitude of outcomes downstream of the interferon response, it is critical we continue to understand the specific roles of IFITM3+ cells in AD.

In conclusion, this wild-derived AD panel offers a level of genetic and phenotypic diversity that can aid in determining the role of microglia in human AD. There will be continued debate regarding the level at which the mouse immune system should be "humanized" in order to better model human immune function. However, based on our data, and with improved tools and resources such as strain-specific gene editing protocols and reporter and Cre lines, integrating the use of wild-derived strains appears essential to more closely align mouse studies to human AD.

STAR★METHODS

Detailed methods are provided in the online version of this paper and include the following:

- KEY RESOURCE TABLE
- RESOURCE AVAILABILITY
  - Lead contact
  - Materials availability
  - Data and code availability
- EXPERIMENTAL MODEL AND SUBJECT DETAILS
  - Ethics statement
  - Mouse strains and cohort generation
- METHOD DETAILS
  - Single myeloid cell preparation
  - Single-cell library preparation and RNA-sequencing
  - Gene expression quantification from scRNA-seq data
  - Identification of brain myeloid cell types and microglia subtypes
  - Differential gene expression and marker gene identification of microglia subclusters
  - Comparison of published mouse and human microglia states/module and enrichment analysis
  - Pseudotime analysis
  - Ingenuity pathway analysis (IPA)
  - Human AD-relevant GWAS gene selection
- QUANTIFICATION AND STATISTICAL ANALYSIS

Supplemental information

Supplemental information can be found online at https://doi.org/10.1016/j.celrep.2021.108739.

ACKNOWLEDGMENTS

We gratefully acknowledge the contribution of Sandy Daigle, Michael Samuels, and Paul Robson at the Single Cell Biology Laboratory at The Jackson Laboratory (JAX) for expert assistance with this publication. We thank Duy Pham from the Churchill lab at JAX for advice on pre-processing of scRNA-seq data on high-performance computing cluster. We thank Christoph Preuss from the Carter lab at JAX for advice on analysis using human AD-relevant GWAS genes. We thank Sandeep Namburi at JAX research IT for installing essential packages and troubleshooting on RStudio server. We thank Jane Cha from JAX Creative for the design and creation of our graphical abstract. This study was supported in part by the National Institute of Aging RF1AG051496 (G.R.H., H.S.Y., and K.D.O.) and RF1AG055104 (G.R.H. and K.D.O.), the Alzheimer's Association Research Fellowship 2018-AARF-589154 (H.S.Y.), and ongoing philanthropic support from the Diana Davis Spencer Foundation (G.R.H.).

AUTHOR CONTRIBUTIONS

H.S.Y., K.D.O., and G.R.H. designed the study. H.S.Y. developed experimental protocols, prepared samples for scRNA-seq, and performed bioinformatics analysis. K.D.O. and K.J.K. generated and maintained mice for this study. K.C. built the pipeline for the quantification of gene expression for the scRNA-seq data. K.C., D.A.S., and G.W.C. provided advice on the scRNA-seq data analysis. K.D.O., H.S.Y., and G.R.H. wrote the manuscript. All authors approved the final version.

DECLARATION OF INTERESTS

The authors declare no competing interests.

Received: June 26, 2020
Revised: November 9, 2020
Accepted: January 15, 2021
Published: February 9, 2021

REFERENCES

Abdul-Sater, A.A., Saïd-Sadier, N., Lam, V.M., Singh, B., Pettengill, M.A., Soares, F., Tattoli, I., Lipinski, S., Girardin, S.E., Rosenstiel, P., and Ojcius, D.M. (2010). Enhancement of reactive oxygen species production and chlamydial infection by the mitochondrial Nod-like family member NLRX1. J. Biol. Chem. *285*, 41637–41645.

Adriouch, S., Dox, C., Welge, V., Seman, M., Koch-Nolte, F., and Haag, F. (2002). Cutting edge: a natural P451L mutation in the cytoplasmic domain impairs the function of the mouse P2X7 receptor. J. Immunol. *169*, 4108–4112.

Agora Consortium (2020). Agora.https://agora.ampadportal.org/genes.

Alsema, A.M., Jiang, Q., Kracht, L., Gerrits, E., Dubbelaar, M.L., Miedema, A., Brouwer, N., Woodbury, M., Wachter, A., Xi, H.S., et al. (2020). Profiling microglia from AD donors and non-demented elderly in acute human post-mortem cortical tissue. bioRxiv. https://doi.org/10.1101/2020.03.18.995332.

Angerer, P., Haghverdi, L., Büttner, M., Theis, F.J., Marr, C., and Buettner, F. (2016). destiny: diffusion maps for large-scale single-cell data in R. Bioinformatics *32*, 1241–1243.

Bohlen, C.J., Bennett, F.C., and Bennett, M.L. (2019). Isolation and Culture of Microglia. Curr. Protoc. Immunol. *125*, e70.

Boza-Serrano, A., Yang, Y., Paulus, A., and Deierborg, T. (2018). Innate immune alterations are elicited in microglial cells before plaque deposition in the Alzheimer's disease mouse model 5xFAD. Sci. Rep. *8*, 1550.

Butler, A., Hoffman, P., Smibert, P., Papalexi, E., and Satija, R. (2018). Integrating single-cell transcriptomic data across different conditions, technologies, and species. Nat. Biotechnol. *36*, 411–420.

Butovsky, O., and Weiner, H.L. (2018). Microglial signatures and their role in health and disease. Nat. Rev. Neurosci. *19*, 622–635.

Calandria, J.M., Asatryan, A., Balaszczuk, V., Knott, E.J., Jun, B.K., Mukherjee, P.K., Belayev, L., and Bazan, N.G. (2015). NPD1-mediated stereoselective regulation of BIRC3 expression through cREL is decisive for neural cell survival. Cell Death Differ. *22*, 1363–1377.

Cantarella, G., Di Benedetto, G., Puzzo, D., Privitera, L., Loreto, C., Saccone, S., Giunta, S., Palmeri, A., and Bernardini, R. (2015). Neutralization of TNFSF10 ameliorates functional outcome in a murine model of Alzheimer's disease. Brain *138*, 203–216.

Caraci, F., Spampinato, S., Sortino, M.A., Bosco, P., Battaglia, G., Bruno, V., Drago, F., Nicoletti, F., and Copani, A. (2012). Dysfunction of TGF-β1 signaling in Alzheimer's disease: perspectives for neuroprotection. Cell Tissue Res. *347*, 291–301.

Chen, Y., Lun, A.T., and Smyth, G.K. (2016). From reads to genes to pathways: differential expression analysis of RNA-Seq experiments using Rsubread and the edgeR quasi-likelihood pipeline. F1000Res. *5*, 1438.

Chintapaludi, S.R., Uyar, A., Jackson, H.M., Acklin, C.J., Wang, X., Sasner, M., Carter, G.W., and Howell, G.R. (2020). Staging Alzheimer's Disease in the Brain and Retina of B6.APP/PS1 Mice by Transcriptional Profiling. J. Alzheimers Dis. *73*, 1421–1434.

Choi, K., Raghupathy, N., and Churchill, G.A. (2019). A Bayesian mixture model for the analysis of allelic expression in single cells. Nat. Commun. *10*, 5188.

Conway, J.R., Lex, A., and Gehlenborg, N. (2017). UpSetR: an R package for the visualization of intersecting sets and their properties. Bioinformatics *33*, 2938–2940.

Costa, A.S., Agostini, S., Guerini, F.R., Mancuso, R., Zanzottera, M., Ripamonti, E., Racca, V., Nemni, R., and Clerici, M. (2017). Modulation of Immune Responses to Herpes Simplex Virus Type 1 by IFNL3 and IRF7 Polymorphisms: A Study in Alzheimer's Disease. J. Alzheimers Dis. *60*, 1055–1063.

De Rojas, I., Moreno-Grau, S., Tesi, N., Grenier-Boley, B., Andrade, V., Jansen, I., Pedersen, N.L., Stringa, N., Zettergren, A., Hernández, I., et al. (2020). Common variants in Alzheimer's disease: Novel association of six genetic variants with AD and risk stratification by polygenic risk scores. medRxiv. https://doi.org/10.1101/19012021.

Earl, P.L., Americo, J.L., and Moss, B. (2012). Lethal monkeypox virus infection of CAST/EiJ mice is associated with a deficient gamma interferon response. J. Virol. *86*, 9105–9112.

Friedman, B.A., Srinivasan, K., Ayalon, G., Meilandt, W.J., Lin, H., Huntley, M.A., Cao, Y., Lee, S.H., Haddick, P.C.G., Ngu, H., et al. (2018). Diverse Brain Myeloid Expression Profiles Reveal Distinct Microglial Activation States and Aspects of Alzheimer's Disease Not Evident in Mouse Models. Cell Rep. *22*, 832–847.

Geirsdottir, L., David, E., Keren-Shaul, H., Weiner, A., Bohlen, S.C., Neuber, J., Balic, A., Giladi, A., Sheban, F., Dutertre, C.A., et al. (2019). Cross-Species Single-Cell Analysis Reveals Divergence of the Primate Microglia Program. Cell *179*, 1609–1622.e16.

Gomez-Nicola, D., and Perry, V.H. (2015). Microglial dynamics and role in the healthy and diseased brain: a paradigm of functional plasticity. Neuroscientist *21*, 169–184.

Gosselin, D., Skola, D., Coufal, N.G., Holtman, I.R., Schlachetzki, J.C.M., Sajti, E., Jaeger, B.N., O'Connor, C., Fitzpatrick, C., Pasillas, M.P., et al. (2017). An environment-dependent transcriptional network specifies human microglia identity. Science *356*, eaal2332.

Gray, F., Scaravilli, F., Everall, I., Chretien, F., An, S., Boche, D., Adle-Biassette, H., Wingertsmann, L., Durigon, M., Hurtrel, B., et al. (1996). Neuropathology of early HIV-1 infection. Brain Pathol. *6*, 1–15.

Greenberg, S.M., Bacskai, B.J., Hernandez-Guillamon, M., Pruzin, J., Sperling, R., and van Veluw, S.J. (2020). Cerebral amyloid angiopathy and Alzheimer disease - one peptide, two pathways. Nat. Rev. Neurol. *16*, 30–42.

Hammond, T.R., Dufort, C., Dissing-Olesen, L., Giera, S., Young, A., Wysoker, A., Walker, A.J., Gergits, F., Segel, M., Nemesh, J., et al. (2019). Single-Cell RNA Sequencing of Microglia throughout the Mouse Lifespan and in the Injured Brain Reveals Complex Cell-State Changes. Immunity *50*, 253–271.e6.

Hur, J.Y., Frost, G.R., Wu, X., Crump, C., Pan, S.J., Wong, E., Barros, M., Li, T., Nie, P., Zhai, Y., et al. (2020). The innate immunity protein IFITM3 modulates γ-secretase in Alzheimer's disease. Nature *586*, 735–740.

Itzhaki, R.F., Golde, T.E., Heneka, M.T., and Readhead, B. (2020). Do infections have a role in the pathogenesis of Alzheimer disease? Nat. Rev. Neurol. *16*, 193–197.

Jackson, H.M., Soto, I., Graham, L.C., Carter, G.W., and Howell, G.R. (2013). Clustering of transcriptional profiles identifies changes to insulin signaling as an early event in a mouse model of Alzheimer's disease. BMC Genomics *14*, 831.

Jansen, I.E., Savage, J.E., Watanabe, K., Bryois, J., Williams, D.M., Steinberg, S., Sealock, J., Karlsson, I.K., Hägg, S., Athanasiu, L., et al. (2019). Genome-wide meta-analysis identifies new loci and functional pathways influencing Alzheimer's disease risk. Nat. Genet. *51*, 404–413.

Johnson, E.C.B., Dammer, E.B., Duong, D.M., Ping, L., Zhou, M., Yin, L., Higginbotham, L.A., Guajardo, A., White, B., Troncoso, J.C., et al. (2020). Large-scale proteomic analysis of Alzheimer's disease brain and cerebrospinal fluid reveals early changes in energy metabolism associated with microglia and astrocyte activation. Nat. Med. *26*, 769–780.

Kang, S.S., Ebbert, M.T.W., Baker, K.E., Cook, C., Wang, X., Sens, J.P., Kocher, J.P., Petrucelli, L., and Fryer, J.D. (2018). Microglial translational profiling reveals a convergent APOE pathway from aging, amyloid, and tau. J. Exp. Med. *215*, 2235–2245.

Kenney, A.D., McMichael, T.M., Imas, A., Chesarino, N.M., Zhang, L., Dorn, L.E., Wu, Q., Alfaour, O., Amari, F., Chen, M., et al. (2019). IFITM3 protects the heart during influenza virus infection. Proc. Natl. Acad. Sci. USA *116*, 18607–18612.

Keren-Shaul, H., Spinrad, A., Weiner, A., Matcovitch-Natan, O., Dvir-Szternfeld, R., Ulland, T.K., David, E., Baruch, K., Lara-Astaiso, D., Toth, B., et al. (2017). A Unique Microglia Type Associated with Restricting Development of Alzheimer's Disease. Cell *169*, 1276–1290.e17.

Kutz, S.M., Higgins, C.E., and Higgins, P.J. (2012). Novel Combinatorial Therapeutic Targeting of PAI-1 (SERPINE1) Gene Expression in Alzheimer's Disease. Mol. Med. Ther. *1*, 106.

Langmead, B., Trapnell, C., Pop, M., and Salzberg, S.L. (2009). Ultrafast and memory-efficient alignment of short DNA sequences to the human genome. Genome Biol. *10*, R25.

Leist, S.R., Pilzner, C., van den Brand, J.M., Dengler, L., Geffers, R., Kuiken, T., Balling, R., Kollmus, H., and Schughart, K. (2016). Influenza H3N2 infection of the collaborative cross founder strains reveals highly divergent host responses and identifies a unique phenotype in CAST/EiJ mice. BMC Genomics *17*, 143.

Lou, N., Takano, T., Pei, Y., Xavier, A.L., Goldman, S.A., and Nedergaard, M. (2016). Purinergic receptor P2RY12-dependent microglial closure of the injured blood-brain barrier. Proc. Natl. Acad. Sci. USA *113*, 1074–1079.

Mathys, H., Davila-Velderrain, J., Peng, Z., Gao, F., Mohammadi, S., Young, J.Z., Menon, M., He, L., Abdurrob, F., Jiang, X., et al. (2019). Single-cell transcriptomic analysis of Alzheimer's disease. Nature *570*, 332–337.

McCarthy, D.J., Chen, Y., and Smyth, G.K. (2012). Differential expression analysis of multifactor RNA-Seq experiments with respect to biological variation. Nucleic Acids Res. *40*, 4288–4297.

McQuade, A., and Blurton-Jones, M. (2019). Microglia in Alzheimer's Disease: Exploring How Genetics and Phenotype Influence Risk. J. Mol. Biol. *431*, 1805–1817.

Oakley, H., Cole, S.L., Logan, S., Maus, E., Shao, P., Craft, J., Guillozet-Bongaarts, A., Ohno, M., Disterhoft, J., Van Eldik, L., et al. (2006). Intraneuronal beta-amyloid aggregates, neurodegeneration, and neuron loss in transgenic mice with five familial Alzheimer's disease mutations: potential factors in amyloid plaque formation. J. Neurosci. *26*, 10129–10140.

Olah, M., Patrick, E., Villani, A.C., Xu, J., White, C.C., Ryan, K.J., Piehowski, P., Kapasi, A., Nejad, P., Cimpean, M., et al. (2018). A transcriptomic atlas of aged human microglia. Nat. Commun. *9*, 539.

Onos, K.D., Uyar, A., Keezer, K.J., Jackson, H.M., Preuss, C., Acklin, C.J., O'Rourke, R., Buchanan, R., Cossette, T.L., Sukoff Rizzo, S.J., et al. (2019). Enhancing face validity of mouse models of Alzheimer's disease with natural genetic variation. PLoS Genet. *15*, e1008155.

Radde, R., Bolmont, T., Kaeser, S.A., Coomaraswamy, J., Lindau, D., Stoltze, L., Calhoun, M.E., Jäggi, F., Wolburg, H., Gengler, S., et al. (2006). Abeta42-driven cerebral amyloidosis in transgenic mice reveals early and robust pathology. EMBO Rep. *7*, 940–946.

Raghupathy, N., Choi, K., Vincent, M.J., Beane, G.L., Sheppard, K.S., Munger, S.C., Korstanje, R., Pardo-Manual de Villena, F., and Churchill, G.A. (2018). Hierarchical analysis of RNA-seq reads improves the accuracy of allele-specific expression. Bioinformatics *34*, 2177–2184.

Rangaraju, S., Dammer, E.B., Raza, S.A., Rathakrishnan, P., Xiao, H., Gao, T., Duong, D.M., Pennington, M.W., Lah, J.J., Seyfried, N.T., and Levey, A.I.

(2018). Identification and therapeutic modulation of a pro-inflammatory subset of disease-associated-microglia in Alzheimer's disease. Mol. Neurodegener. *13*, 24.

Riphagen, J.M., Ramakers, I.H.G.M., Freeze, W.M., Pagen, L.H.G., Hanseeuw, B.J., Verbeek, M.M., Verhey, F.R.J., and Jacobs, H.I.L. (2020). Linking APOE-ε4, blood-brain barrier dysfunction, and inflammation to Alzheimer's pathology. Neurobiol. Aging *85*, 96–103.

Robinson, M.D., McCarthy, D.J., and Smyth, G.K. (2010). edgeR: a Bioconductor package for differential expression analysis of digital gene expression data. Bioinformatics *26*, 139–140.

Romagnoli, M., Porcellini, E., Carbone, I., Veerhuis, R., and Licastro, F. (2020). Impaired Innate Immunity Mechanisms in the Brain of Alzheimer's Disease. Int. J. Mol. Sci. *21*, 1126.

Roy, E.R., Wang, B., Wan, Y.W., Chiu, G., Cole, A., Yin, Z., Propson, N.E., Xu, Y., Jankowsky, J.L., Liu, Z., et al. (2020). Type I interferon response drives neuroinflammation and synapse loss in Alzheimer disease. J. Clin. Invest. *130*, 1912–1930.

Sala Frigerio, C., Wolfs, L., Fattorelli, N., Thrupp, N., Voytyuk, I., Schmidt, I., Mancuso, R., Chen, W.T., Woodbury, M.E., Srivastava, G., et al. (2019). The Major Risk Factors for Alzheimer's Disease: Age, Sex, and Genes Modulate the Microglia Response to Aβ Plaques. Cell Rep. *27*, 1293–1306.e6.

Sharma, P., and Ping, L. (2014). Calcium ion influx in microglial cells: physiological and therapeutic significance. J. Neurosci. Res. *92*, 409–423.

Sierksma, A., Lu, A., Mancuso, R., Fattorelli, N., Thrupp, N., Salta, E., Zoco, J., Blum, D., Buée, L., De Strooper, B., and Fiers, M. (2020). Novel Alzheimer risk genes determine the microglia response to amyloid-β but not to TAU pathology. EMBO Mol. Med. *12*, e10606.

Spangenberg, E., Severson, P.L., Hohsfield, L.A., Crapser, J., Zhang, J., Burton, E.A., Zhang, Y., Spevak, W., Lin, J., Phan, N.Y., et al. (2019). Sustained microglial depletion with CSF1R inhibitor impairs parenchymal plaque development in an Alzheimer's disease model. Nat. Commun. *10*, 3758.

Stuart, T., and Satija, R. (2019). Integrative single-cell analysis. Nat. Rev. Genet. *20*, 257–272.

Terrien, J., Seugnet, I., Seffou, B., Herrero, M.J., Bowers, J., Chamas, L., Decherf, S., Duvernois-Berthet, E., Djediat, C., Ducos, B., et al. (2019). Reduced central and peripheral inflammatory responses and increased mitochondrial activity contribute to diet-induced obesity resistance in WSB/EiJ mice. Sci. Rep. *9*, 19696.

Thei, L., Imm, J., Kaisis, E., Dallas, M.L., and Kerrigan, T.L. (2018). Microglia in Alzheimer's Disease: A Role for Ion Channels. Front. Neurosci. *12*, 676.

Thrupp, N., Sala Frigerio, C., Wolfs, L., Skene, N.G., Fattorelli, N., Poovathingal, S., Fourne, Y., Matthews, P.M., Theys, T., Mancuso, R., et al. (2020). Single-Nucleus RNA-Seq Is Not Suitable for Detection of Microglial Activation Genes in Humans. Cell Rep. *32*, 108189.

Wu, Y.E., Pan, L., Zuo, Y., Li, X., and Hong, W. (2017). Detecting Activated Cell Populations Using Single-Cell RNA-Seq. Neuron *96*, 313–329.e6.

Yang, H., Graham, L.C., Reagan, A.M., Grabowska, W.A., Schott, W.H., and Howell, G.R. (2019). Transcriptome profiling of brain myeloid cells revealed activation of Itgal, Trem1, and Spp1 in western diet-induced obesity. J. Neuroinflammation *16*, 169.

Zambrano, A., Otth, C., Mujica, L., Concha, I.I., and Maccioni, R.B. (2007). Interleukin-3 prevents neuronal death induced by amyloid peptide. BMC Neurosci. *8*, 82.

Zhang, X., Lan, Y., Xu, J., Quan, F., Zhao, E., Deng, C., Luo, T., Xu, L., Liao, G., Yan, M., et al. (2019). CellMarker: a manually curated resource of cell markers in human and mouse. Nucleic Acids Res. *47* (*D1*), D721–D728.

Zhou, Y., Song, W.M., Andhey, P.S., Swain, A., Levy, T., Miller, K.R., Poliani, P.L., Cominelli, M., Grover, S., Gilfillan, S., et al. (2020). Human and mouse single-nucleus transcriptomics reveal TREM2-dependent and TREM2-independent cellular responses in Alzheimer's disease. Nat. Med. *26*, 131–142.

STAR★METHODS

KEY RESOURCE TABLE

REAGENT or RESOURCE	SOURCE	IDENTIFIER
**Chemicals, peptides, and recombinant proteins**		
HBSS (10X)	ThermoFisher Scientific	Cat#14185-052
HEPES	ThermoFisher Scientific	Cat#15630080
Glucose solution	Sigma-Aldrich	Cat#49163
DNase I	Worthington	Cat#DPRFS
FBS	ThermoFisher Scientific	Cat#16000069
BSA	Jackson ImmunoResearch	Cat#001-000-173
Ultrapure EDTA	ThermoFisher Scientific	Cat#15575020
PBS (10X)	ThermoFisher Scientific	Cat#70011044
Trypan Blue Stain (0.4%)	ThermoFisher Scientific	Cat#T10282
**Critical commercial assays**		
Chromium Single Cell 3′ Reagent Kits	10X Genomics	Cat#CG00052
CD11b Microglia Microbeads	Miltenyi Biotec	Cat#130-049-601
Myelin removal Beads II	Miltenyi Biotec	Cat#130-096-733
**Deposited data**		
Raw and processed data and metadata spreadsheet	This paper	https://doi.org/10.7303/syn23763409
Shiny app for searching B6 and wild microglia database	This paper	https://wild_microglia_scrna-seq.jax.org/
Code for reproducing the analysis	This paper	https://github.com/TheJacksonLaboratory/wild_AD_mic_scRNA
scRNA-seq data of microglia from 5xFAD mice (C57BL/6J and SJL/J background)	Keren-Shaul et al., 2017	https://www.cell.com/fulltext/S0092-8674(17)30578-0
scRNA-seq data of microglia from *App*$^{NL-G-F}$ mice (C57BL/6J background)	Sala Frigerio et al., 2019	GEO: GSE127893
scRNA-seq data of mouse microglia throughout lifespan (C57BL/6J background)	Hammond et al., 2019	http://www.microgliasinglecell.com/
snRNA-seq data of human AD patients with TREM2 variants	Zhou et al., 2020	https://www.nature.com/articles/s41591-019-0695-9
snRNA-seq data of human AD patients	Mathys et al., 2019	https://www.nature.com/articles/s41586-019-1195-2
RNA-seq data of aged human microglia	Olah et al., 2018	https://www.nature.com/articles/s41467-018-02926-5
proteomic analysis of Alzheimer's disease brain and cerebrospinal fluid	Johnson et al., 2020	https://www.nature.com/articles/s41591-020-0815-6
**Experimental models: organisms/strains**		
B6.Cg-Tg(APPswe, PSEN1dE9)85Dbo/Mmjax	The Jackson Laboratory	Stock#005864
CAST.*APP/PS1*	The Jackson Laboratory	Stock#25973
PWK.*APP/PS1*	The Jackson Laboratory	Stock#25971
WSB.*APP/PS1*	The Jackson Laboratory	Stock#25970
**Software and algorithms**		
scBASE	Choi et al., 2019	https://hub.docker.com/r/kbchoi/asesuite-sc
bcl2fastq (v 2.20.0.422)	Illumina	https://support.illumina.com/sequencing/sequencing_software/bcl2fastq-conversion-software.html
Seurat (v 3.1.2)	Stuart and Satija, 2019; Butler et al., 2018	https://satijalab.org/seurat/

(*Continued on next page*)

Continued

REAGENT or RESOURCE	SOURCE	IDENTIFIER
edgeR (v 3.28.0)	Chen et al., 2016; Robinson et al., 2010; McCarthy et al., 2012	https://bioconductor.org/packages/release/bioc/html/edgeR.html
R (v 3.6.0)	R Core Team	https://www.r-project.org/
RStudio Server Pro (1.3.1056-1)	RStudio	https://rstudio.com/
Ingenuity Pathway Analysis	QIAGEN	NA
UpSetR (v 1.4.0)	Conway et al., 2017	https://github.com/hms-dbmi/UpSetR
Others		
MACS SmartStrainer (70 μm)	Miltenyi Biotec	Cat#130-098-462
LD columns	Miltenyi Biotec	Cat#130-042-901
LS columns	Miltenyi Biotec	Cat#130-042-401
Supreme Mini-treats	BioServ	Cat#F05472 or F05711

RESOURCE AVAILABILITY

Lead contact

Further information and requests for resources and reagents should be directed to and will be fulfilled by the lead contact, Gareth Howell (gareth.howell@jax.org).

Materials availability

All mouse strains are available through The Jackson Laboratory. All reagents in this study are commercially available.

Data and code availability

The raw data, processed data, and sample information in the study are available via the AD Knowledge Portal (http://adknowledgeportal.synapse.org). The AD Knowledge Portal is a platform for accessing data, analyses, and tools generated by the Accelerating Medicines Partnership (AMP-AD) Target Discovery Program and other National Institute on Aging (NIA)-supported programs to enable open-science practices and accelerate translational learning. The data, analyses and tools are shared early in the research cycle without a publication embargo on secondary use. Data is available for general research use according to the following requirements for data access and data attribution (http://adknowledgeportal.synapse.org/DataAccess/Instructions). For access to content described in this manuscript see: https://doi.org/10.7303/syn23763409. All the code for the data analysis is available at JAX Github repository (https://github.com/TheJacksonLaboratory/wild_AD_mic_scRNA). A shiny app for querying microglia genes is available at (https://wild_microglia_scrna-seq.jax.org/).

EXPERIMENTAL MODEL AND SUBJECT DETAILS

Ethics statement

All research was approved by the Institutional Animal Care and Use Committee (IACUC) at The Jackson Laboratory (approval number 12005). Authors performed their work following guidelines established by the "The Eighth Edition of the Guide for the Care and Use of Laboratory Animals" and euthanasia using methods approved by the American Veterinary Medical Association."

Mouse strains and cohort generation

All mice were bred and housed in a 12/12 hours light/dark cycle on aspen bedding and fed standard 6% LabDiet Chow. Experiments were performed on four mouse strains: B6.Cg-Tg(APPswe, PSEN1dE9)85Dbo/Mmjax (JAX stock #005864), CAST.*APP/PS1* (JAX Stock #25973), WSB.*APP/PS1* (JAX Stock #25970) and PWK.*APP/PS1* (JAX Stock #25971). Generation of experimental cohorts consisted of 6 female mice (*APP/PS1* carriers and littermate wild-type controls). Due to increased pup mortality in the wild-derived strains, once determined to be pregnant, female mice were removed from the mating and housed individually. During this time, they were also given BioServ Supreme Mini-treats (Chocolate #F05472 or Very Berry Flavor #F05711) in order to discourage pup cannibalism. Mice were initially group-housed during aging and then individually housed if fighting occurred. Brains were harvested from all mice at 8-9 months of age.

METHOD DETAILS

Single myeloid cell preparation

Four mice were included in each of the B6, B6.*APP/PS1,* CAST, CAST.*APP/PS1,* WSB, and WSB.*APP/PS1* groups (n = 4), five in PWK (n = 5) and three in PWK.*APP/PS1* (n = 3) for initial sample preparation and sequencing. However, two PWK samples and one CAST.*APP/PS1* sample were excluded due to failed execution in scBASE pipeline, resulting in three mice for each of the PWK and CAST.*APP/PS1* groups (n = 3). With modification from the protocol of Bohlen et al. (2019), brain myeloid single-cell suspension were obtained through mechanical dissociation followed by magnetic-activated cell sorting (MACS). All procedures were performed on ice or under 4°C to avoid *ex vivo* activation of microglia during the sample preparation. Mice were anesthetized using ketamine/xylazine (10 mg ketamine and 2 mg xylazine in 0.1ml sterile pure water per 10 g body weight) and perfused using ice cold homogenization buffer [Hank's balanced salt solution (HBSS) containing 15mM HEPES and 0.5% glucose]. Brains were quickly dissected and transferred on ice. Each brain was minced using a scalpel and then homogenized using a 15 mL PTFE tissue grinder (4-5 strokes) in 2mL homogenization buffer containing 320KU /ml DNaseI (Worthington. Cat# DPRFS). The cell suspension was transferred to a 50 mL tube and passed through a pre-wet (with homogenization/DNAase I buffer) 70 micron cell strainer. The filtered cell suspension was then transferred into a 15 mL tube and spun down at 500 g for 5 minutes at 4°C. The supernatant was discarded, and the cell pellet was resuspended in 2 mL MACS buffer [Phosphate-buffered saline (PBS) with 0.5% BSA and 2mM Ultrapure EDTA] for myelin removal procedure. 200 μL Myelin Removal Beads II (Miltenyi Biotec #130-096-733) was added to the cell suspension and mixed gently by pipetting. The cell suspension was then divided into two 2 mL microcentrifuge tubes (1 mL per tube) and incubated for 10 minutes at 4°C. The cell suspension in each tube was diluted up to 2 mL with MACS buffer and centrifuged for 30 s at 9300 g, 4°C. The supernatants were discarded, and the cell pellets were resuspended in 1.5 mL MACS buffer per tube. The cell suspensions from each tube were transferred to two pre-wet LD columns (with MACS buffer, two LD columns for one brain sample, Miltenyi Biotec #130-042-901) and the cell flow-through were collected in 50 mL tubes on ice in a big covered Styrofoam cooler. The LD columns were rinsed twice with 2 mL MACS buffer. The flow-throughs were divided into multiple 2 mL tubes and centrifuged for 30 s at 9300 g, 4°C. The supernatants were discarded, and the cell pellets were resuspended collectively in 1mL PBS for each sample. The brain myeloid cells were enriched by MACS using CD11b MicroBeads (Miltenyi Biotec # 130-049-601) according to manufacturer's instructions. The cell viability was indicated by Trypan Blue and live/dead cell numbers were determined using an automated cell counter. Samples with cell viability more than 80% were subjected to single-cell RNA sequencing.

Single-cell library preparation and RNA-sequencing

MACS-enriched brain myeloid cells were subjected to single-cell library preparation. For each sample approximately 12,000 cells were washed and resuspended in PBS containing 3% FBS and immediately processed as follows. Single-cell capture, barcoding and library preparation were performed using the 10X Chromium platform (10X Genomics), using version 3 chemistry according to the manufacturer's protocol (10X Genomics #CG00052). The resulting cDNA and indexed libraries were checked for quality on an Agilent 4200 TapeStation, quantified by KAPA qPCR, and pooled for sequencing on 16.67% of lane of an Illumina NovaSeq 6000 S2 flow cell, targeting 6,000 barcoded cells with an average sequencing depth of 50,000 reads per cell. Illumina base call (bcl) files for the samples were converted to FASTQ files using CellRanger bcl2fastq (version 2.20.0.422, Illumina).

Gene expression quantification from scRNA-seq data

The analysis pipeline of scBASE (Choi et al., 2019) was used in order to avoid alignment bias due to differences in genetic background of mouse strains. First, we built the read alignment index by combining the custom strain-specific transcriptomes of CAST/EiJ, PWK/PhJ, WSB/EiJ, and C57BL/6J, created with g2gtools (http://churchill-lab.github.io/g2gtools). We removed PCR duplicates from the raw scRNA-seq data, and then aligned the remaining reads to the pooled transcriptome of the four strains using bowtie (Langmead et al., 2009) with '—all', '—best', and '—strata' options. We processed the resulting bam files into an alignment incidence matrix (emase format) using alntools (https://churchill-lab.github.io/alntools) and quantified gene expression for each cell with emase-zero (Raghupathy et al., 2018) (https://github.com/churchill-lab/emase-zero). We collated the estimated UMI counts into a loom formatted file (http://loompy.org) for downstream analysis. A docker container in which all the above-mentioned software tools are pre-installed is freely available at https://hub.docker.com/r/kbchoi/asesuite-sc.

Identification of brain myeloid cell types and microglia subtypes

First, we identified myeloid cell types by filtering out non-myeloid cells using a standard Seurat (v3.1.2) (Butler et al., 2018; Stuart and Satija, 2019) clustering pipeline for each strain, respectively. Cells with fewer than 600 detected genes or higher than 8% of mitochondrial genes were removed before initial analysis. We performed dimension reduction using PCA followed by UMAP using 3,000 most variable genes after normalizing the UMI counts. We identified marker genes for each clusters (FindAllMarkers) with default setting, and then annotated each cluster using enrichCellMarkers package (Zhang et al., 2019). We repeated the same clustering analysis after filtering out non-myeloid cells to refine PCA projection of myeloid cell types for each strain. We integrated myeloid cell clusters across the mouse strains (IntegrateData) and repeated the same clustering analysis. We identified a total of 91,201 myeloid cells including microglia, perivascular macrophage, monocytes and neutrophil (22,212 from B6, 24,976 from CAST; 20,192 from PWK and 23,821 from WSB, Figure S1). Next, for microglia sub-clustering, we selected only those cells defined as microglia

(unintegrated data) for integration and repeated the same clustering analysis. We identified a total of 87,746 microglia composed of 13 putative microglia subtypes (20,732 from B6, 24,124 from CAST, 19,702 from PWK and 23,188 from WSB, Figures S1A–S1D and S2A–S2C).

Differential gene expression and marker gene identification of microglia subclusters

The strain, genotype and strain by genotype effect on single-cell gene expression for each microglia cluster was assessed by edgeR package (Robinson et al., 2010; Chen et al., 2016; McCarthy et al., 2012). The single-cell microglia gene raw counts from a given cluster of each sample was summed as pseudo-bulk gene expression data before passing to standard differentially expressed (DE) gene analysis pipeline of edgeR using a quasi-likelihood method (glmQLFTest function) (http://bioconductor.org/books/release/OSCA/). The gene expression model was built to access the strain, genotype and strain by genotype effect while regressing out batch effect (psedo-bulk gene expression/cluster ~strain + genotype + strain:genotype + batch). The complete DE gene analysis results with all coefficients for each cluster were reported in Table S3 (FDR < 0.05 is considered significant). The initial myeloid and microglia marker genes for each clusters were determined using FindAllMarkers with the default Wilcoxon rank sum test in Seurat package, comparing gene expression of a given cluster to the rest of the clusters with all groups combined (Figures 2, 3, and 4, p.adj < 0.05 was considered significant). For strain-specific microglia marker gene comparison (Figures 4, 5, and S7; Table S5), DAM (cluster 6 and 12), IRM (cluster 7), *Ccl3*^{high}/*Ccl4*^{high} (cluster 10) microglia were compared to homeostatic microglia within each strain (genotype combined) using FindMarkers with Wilcoxon rank sum test in Seurat package.

Comparison of published mouse and human microglia states/module and enrichment analysis

We extensively compared our dataset to publicly available microglia datasets from both mice and human. We extracted the marker gene sets of previously identified aging or AD-relevant microglia states from mice (B6 background): disease-associated microglia (DAM, see Table S3 in Keren-Shaul et al., 2017); activated response microglia (ARM), cycling and proliferating microglia (CPM) and interferon-response microglia (IRM) reproduced using meta data spreadsheet and count matrix from GSE127893 (Sala Frigerio et al., 2019); Aging OA2 and OA3 microglia (Hammond et al., 2019). We also extracted the marker sets of previously determined aging or AD-relevant microglia states/module in human: Mic1 population from Table S7 (Mathys et al., 2019); Micro0 population from Table S4 (Zhou et al., 2020); aged microglia from Supplemental Data 1 (Olah et al., 2018); M4 microglia module from Table S5 (Johnson et al., 2020). To evaluate the enrichment of mark gene sets in each microglial cells in our dataset, we calculated the average expression levels of each gene of marker sets for each cell, subtracted by the aggregated expression of random control gene sets, using Seurat's "AddModuleScore" function. The resulting z-scores were plotted in a violin-boxplot according to clusters, strains, or genotypes.

For cluster annotation (Figure 1E), we selected positive microglia state markers from DAM, ARM, Aging OA2, IRM, Aging OA3, CPM using a stringent threshold [$-\log_{10}$(FDR) > 10 and $\log_2$(FC) > 0.5 and no more than 50 genes ranked by FDR, FDR=false discovery rate, FC=fold change], resulting 49, 27, 50, 35, 31 and 1 marker genes used for enrichment analysis by AddModuleScore, respectively. Because there was only 1 marker gene (*Mcm6*) for CPM detected in our reproduced analysis from the de Strooper study (Sala Frigerio et al., 2019), we included two additional CPM maker genes (*Top2a* and *Mcm2*) as exemplified in the original paper for enrichment analysis. To evaluate homeostatic microglia gene enrichment (Figure 1E) for microglia annotation, we curated a list of 23 classical homeostatic microglial genes based on multiple previous studies (Keren-Shaul et al., 2017; Sala Frigerio et al., 2019; Hammond et al., 2019; Gosselin et al., 2017; Butovsky and Weiner, 2018) including *Tmem119, Cx3cr1, P2ry12, P2ry13, Hexb, Olfml3, Selplg, Siglech, Csf1r, Cst3, Sparc, C1qa, C1qb, C1qc, Tmsb4x, Sall1, Fcrls, Gpr34, Spi1, Mafb, Maf, Mef2a,* and *Irf8*.

We used upset plots (Conway et al., 2017) to visualize the intersections of marker gene sets of microglia subclusters (DAM, IRM, *Ccl3*^{high}/*Ccl4*^{high}) from each strain and the above corresponding mouse microglia states and human Mic1 population (Figures 4A, 5A, 6A, S6A, and S7A). The marker gene sets for each cluster from each strain used in the upset plots are selected using following the criteria: $\log_2$(FC) > 0.25 and $-\log_{10}$(FDR) > 18 for DAM (cluster 6 or 12); $\log_2$(FC) > 0.25 and $-\log_{10}$(FDR) > 8 for IRM (cluster 7) and *Ccl3*^{high}/*Ccl4*^{high} (cluster 10). The lower stringency of $-\log_{10}$(FDR) for IRM and *Ccl3*^{high}/*Ccl4*^{high} microglia allowed enough numbers of marker genes for comparison. A relaxed and varied stringency was applied to the above publicly available mouse and human datasets to include reasonable numbers of maker genes for comparison (due to the differences in data source): $\log_2$(FC) > 0.25 and $-\log_{10}$(FDR) > 18 for DAM; $\log_2$(FC) > 0.25 and $-\log_{10}$(FDR) > 6 for ARM and IRM; $\log_2$(FC) > 0.25 and top 50 genes ranked by FDR for Aging OA2; $\log_2$(FC) > 0.25 and $-\log_{10}$(FDR) > 1.3 for Aging OA3; all marker genes listed for human Mic1; $\log_2$(FC) > 0.5 and $-\log_{10}$(FDR) > 6 for human Micro0; top 50 positive marker genes for aged human microglia; $-\log_{10}$(FDR) > 1.3 and upregulated genes for M4 microglia module.

Pseudotime analysis

We performed pseudotime analysis for microglia using 'destiny' package (Angerer et al., 2016), a diffusion map based-pseudotime inference. Because 'destiny' cannot generate a diffusion map for all 87,746 cells, we randomly sampled 1,000 cells from each group (8000 cells for 8 groups). The first 30 principal components from these cells were processed through the 'dpt' function to generate a diffusion map. The first dimension of the diffusion map was used as the pseudotime axis. A histogram displaying the distribution of 1000 microglia of each group along the pseudotime was plotted, with microglia cluster color coded.

Ingenuity pathway analysis (IPA)

The DE genes (Table S3) comparing wild-derived strains to B6 for homeostatic microglia (cluster H: 0-5 combined), DAM (cluster 6), IRM (cluster 7) and *Ccl3*^{high}/*Ccl4*^{high} microglia (cluster 10) were subjected to Diseases and Functions (DF) and Regulatory Effect (RE) analysis of IPA. The DE genes uploaded to IPA was defined as FDR < 0.05 and |FC| > 2 for homeostatic microglia, |FC| > 1 for DAM, IRM, and *Ccl3*^{high}/*Ccl4*^{high} microglia. The higher FC threshold for homeostatic microglia was because there were too many DE genes in homeostatic microglia when FC was set at 1 which was not computationally efficient for IPA. The top 20 (approximately) most significant terms of DF from any of the comparisons (CAST versus B6, PWK versus B6, WSB versus B6) were visualized in a heatmap.

Human AD-relevant GWAS gene selection

The human AD-relevant GWAS genes were selected from two recent meta-analyses (Table 1 in Jansen et al., 2019 and Table S2 in De Rojas et al., 2020). The GWAS genes from both tables were combined and were overlapped with the homologous mouse genes in scRNA-seq data from this study. These GWAS genes were summarized in Table S6.

QUANTIFICATION AND STATISTICAL ANALYSIS

The proportion of microglia subtypes in each sample was calculated by dividing the number of cells in a given cluster by the total number of cells from each sample. A two-way ANOVA with Tukey's post hoc test (aov and TukeyHSD function in base R) was employed to assess the strain, genotype, and strain by genotype effect on the percent of cells per cluster. Significance for genotype comparisons within strains were reported in each figure (Figure 2C). The complete comparisons with confidence interval and adjusted p values (p.adj) are reported in Table S2. A one-way ANOVA with Tukey's post hoc test (aov and TukeyHSD function in base R) was employed to assess the enrichment z-score difference across clusters for a given gene set enrichment (Figure 1E). A two-way ANOVA with Tukey's post hoc test was employed to assess the strain, genotype, and strain by genotype effect on the z-score for each cluster (Figures 3A, 4B, 5B, 6B, S6B, and S7B). Significance of the comparisons were reported in each figure.

Cell Reports, Volume *34*

Supplemental Information

Natural genetic variation determines microglia heterogeneity in wild-derived mouse models of Alzheimer's disease

Hongtian Stanley Yang, Kristen D. Onos, Kwangbom Choi, Kelly J. Keezer, Daniel A. Skelly, Gregory W. Carter, and Gareth R. Howell

Figure S1

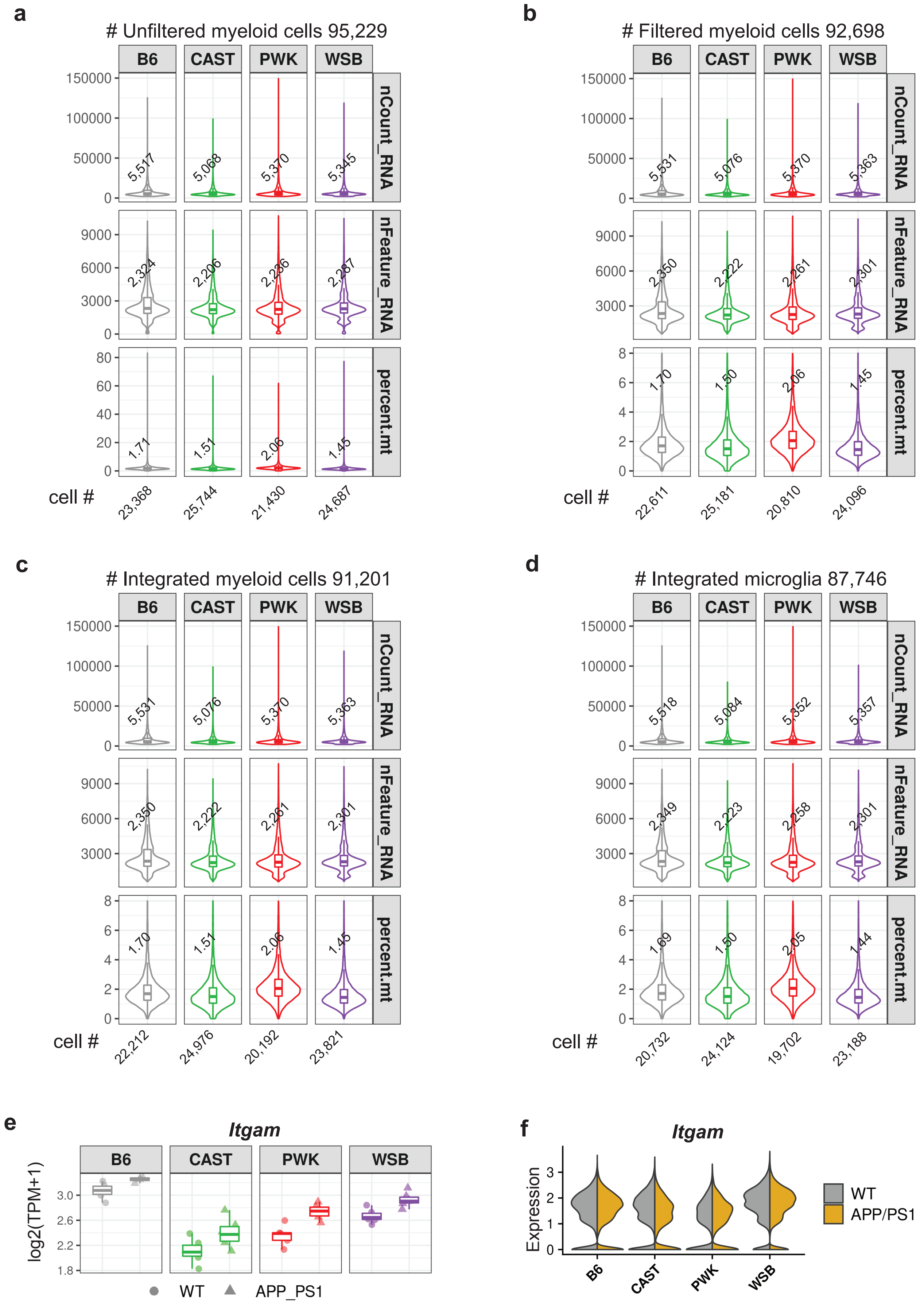

a
Unfiltered myeloid cells 95,229
B6
CAST
PWK
WSB
nCount_RNA
nFeature_RNA
percent.mt
5,517
5,068
5,370
5,345
2,324
2,206
2,236
2,287
1.71
1.51
2.06
1.45
cell #
23,368
25,744
21,430
24,687
b
Filtered myeloid cells 92,698
5,531
5,076
5,370
5,363
2,350
2,222
2,261
2,301
1.70
1.50
2.06
1.45
cell #
22,611
25,181
20,810
24,096
c
Integrated myeloid cells 91,201
5,531
5,076
5,370
5,363
2,350
2,222
2,261
2,301
1.70
1.51
2.06
1.45
cell #
22,212
24,976
20,192
23,821
d
Integrated microglia 87,746
5,518
5,084
5,352
5,357
2,349
2,223
2,258
2,301
1.69
1.50
2.05
1.44
cell #
20,732
24,124
19,702
23,188
e
Itgam
log2(TPM+1)
WT
APP_PS1
f
Itgam
Expression
WT
APP/PS1

Fig. S1 | Quality control for single-cell RNA-seq, Related to Fig.1. a-d, Violin-box plots showing the distribution of the number of total RNA reads (nCount_RNA), the total number of genes detected (nFeature_RNA) and the percent mitochondrial genes in individual cells from each strain after each quality control steps. The medians were labeled for each plot. **a**, Recovered cells after gene expression quantification by emaze-zero. **b**, Remaining cells after removing cells with high-percent mitochondrial genes (>8%). **c**, Remaining cells after myeloid cell integration based on strain. Cells that were not integrated likely did not contain sufficient anchor features as required by the *FindTransferAnchors* and *Transferdata* functions in the Seurat package. **d**, Remaining cells after microglia integration based on strain. **e**. Box plots showing *Itgam* expression detected from bulk RNA-seq from whole brain of age- and sex-matched (8 month female) mice for each strain and genotype[6].The expression of *Itgam* is significantly affected by strain (FDR= 3.3×10^{-13} , generalized linear regression) but not genotype (*APP/PS1*). **f**. Violin plots showing *Itgam* expression in all myeloid cells after integration (c) for each strain and genotype. The expression of *Itgam* was significantly affected by strain (FDR<0.05, edgeR, wild vs B6) but not genotype.

Figure S2

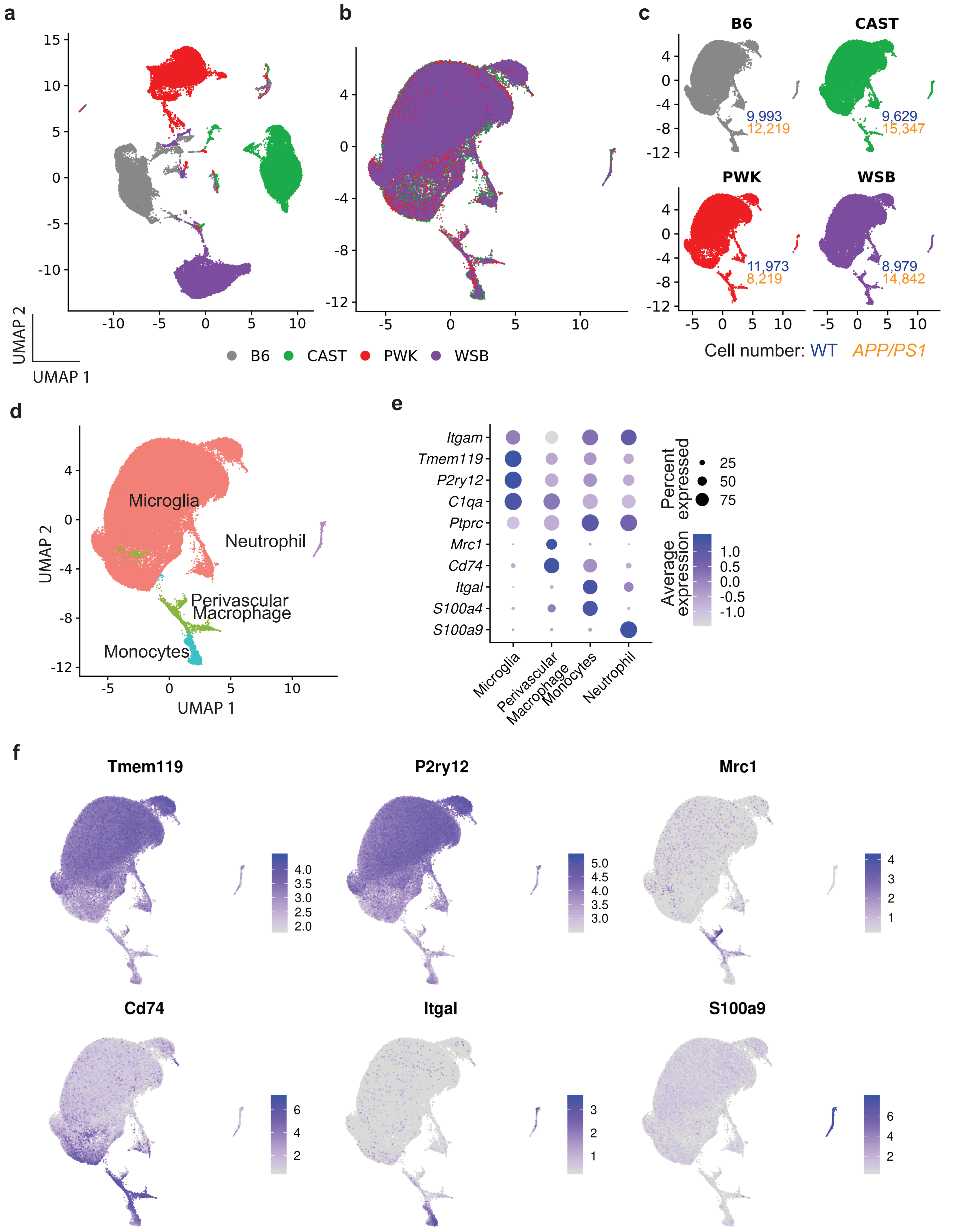
a
b
c
B6
CAST
PWK
WSB
9,993
12,219
9,629
15,347
11,973
8,219
8,979
14,842
Cell number: WT APP/PS1
UMAP 2
UMAP 1
B6 CAST PWK WSB
d
Microglia
Neutrophil
Perivascular Macrophage
Monocytes
e
Itgam
Tmem119
P2ry12
C1qa
Ptprc
Mrc1
Cd74
Itgal
S100a4
S100a9
Microglia
Perivascular Macrophage
Monocytes
Neutrophil
Percent expressed
25
50
75
Average expression
1.0
0.5
0.0
-0.5
-1.0
f
Tmem119
P2ry12
Mrc1
Cd74
Itgal
S100a9

Fig. S2 | A single-cell atlas revealed natural genetic variation shapes the transcriptome of brain myeloid cells, Related to Fig.1. a, Raw cell map of brain myeloid cells in WT and *APP/PS1* of B6, CAST, PWK and WSB mice. UMAP of 92,698 single brain myeloid cell profiles in all groups of mice (n = 3-4/genotype/strain, 29 mice in total), without data integration on the strain. **b**-**c**, Strain-integrated cell map of brain myeloid cells of WT and *APP/PS1* of B6, CAST, PWK and WSB mice. UMAP of 91,201 single brain myeloid cell profiles in all groups of mice with merged view (b) and split views based on strain (c), with canonical correlation analysis (CCA)-based integration on strains. **d**, Single-cell profiling revealed 4 major distinct myeloid cell types including microglia, perivascular macrophages, monocytes and neutrophils. The integrated UMAP showing 91,201 single brain myeloid cell profiles in all groups (29 mice) colored by myeloid cell types. **e**, Dot plot showing the classical marker genes for myeloid cell types with their percent expressed and average expression. **f**, UMAP plots featuring cluster-defining marker genes including *Tmem119*, *P2ry12*, *Mrc1*, *Cd74*, *Itgal* and *S100a9*.

Figure S3

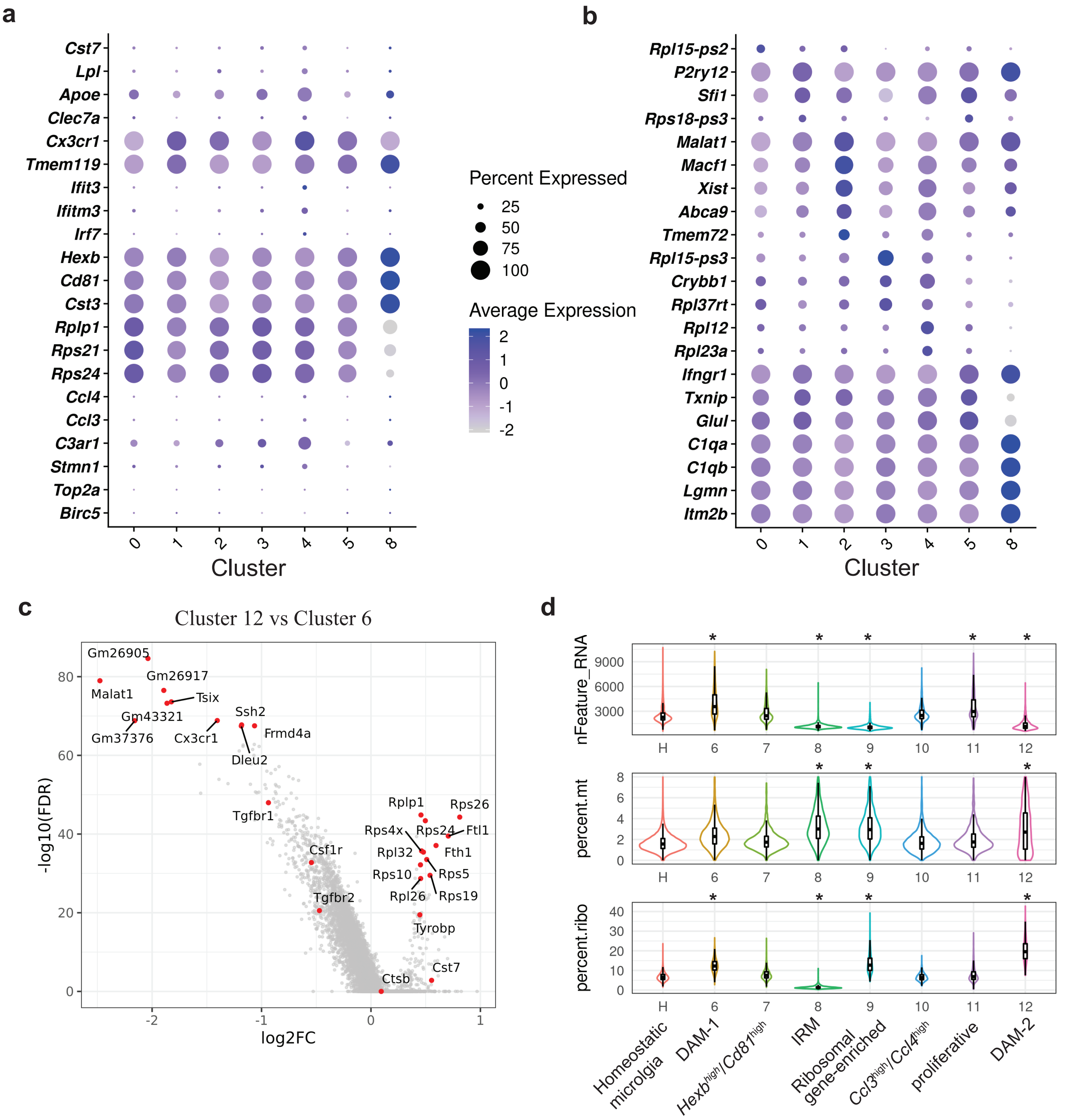

Fig. S3 | Additional features of microglia subtypes, Related to Fig.2. a, Dot plot showing the expression of top microglia marker genes of all clusters (Fig. 1b) in homeostatic microglia subtypes (clusters 0, 1, 2, 3, 4, 5) and *Hexb*^{high}/*Cd81*^{high} microglia (cluster 8). **b**, Dot plot showing the expression of the top microglia marker genes in homeostatic microglia (clusters 0,1, 2, 3, 4, 5) and 8. **c**, Volcano plot showing the top up- and down- regulated genes comparing DAM cluster 12 to DAM cluster 6. Cluster 12 showed increased expression in ribosomal genes and *Tyrobp* (log2FC > 0) and decreased expression in homeostatic microglia genes including *Cx3cr1*, *Tgfbr1*, *Csf1r* and *Tgfbr2* (log_2FC < 0). **d**, Violin-box plots showing the distribution of the total number of genes detected (nFeature_RNA), the percent of mitochondrial (percent.mt) and ribosomal (percent.ribo) genes in cells from all identified microglia subtypes. * adjusted p value (p.adj) < 0.05. All comparisons were made by comparing the medians in clusters 6, 7, 8, 9, 10, 11 and 12 to combined homeostatic microglia (cluster 0 to 5), respectively, using one-way ANOVA followed by Tukey's post hoc test.

Figure S4

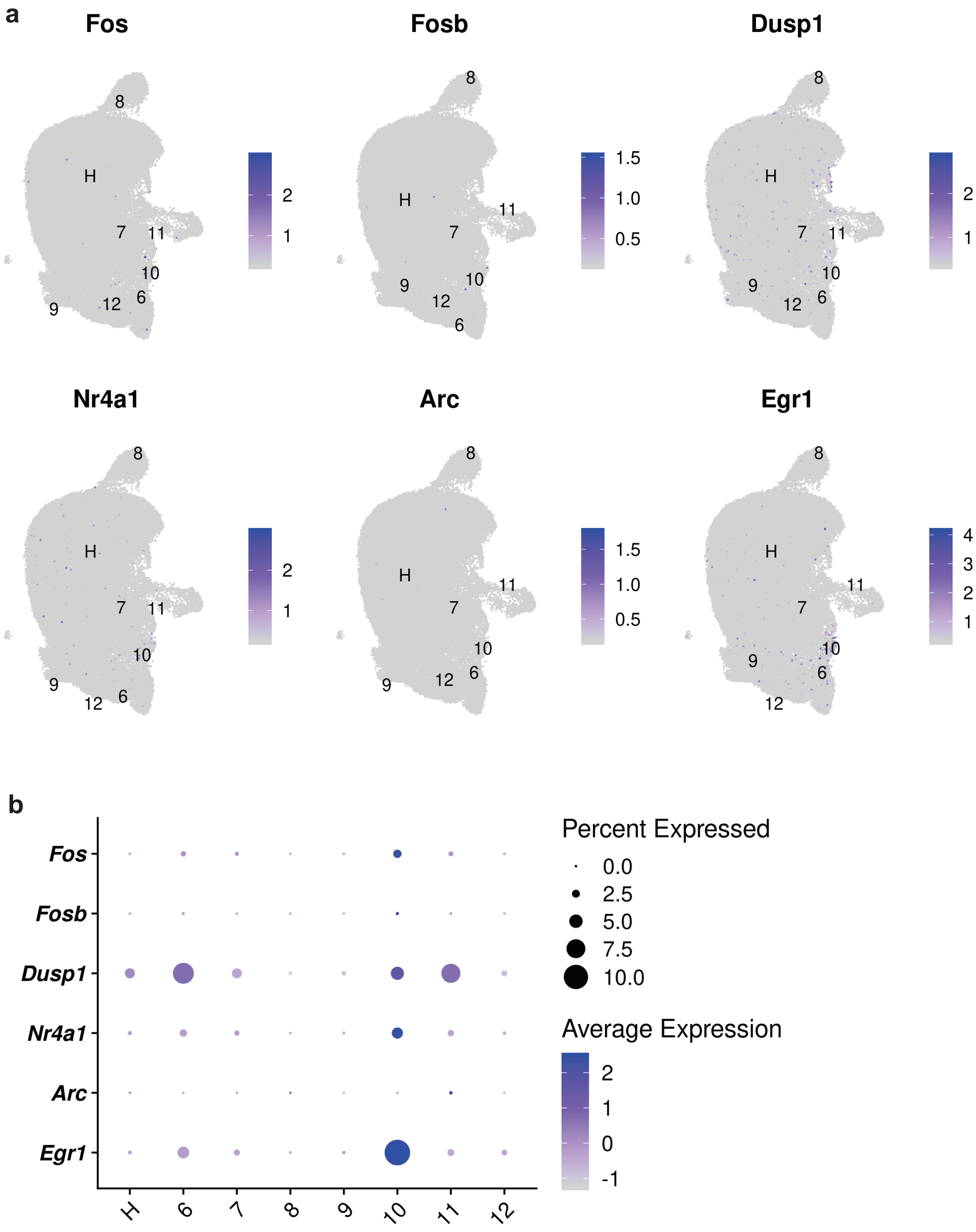

Fig. S4 | Immediate early gene expression in microglia subclusters, Related to Fig.1. a-b, UMAP plots (a) and dot plot (b) showing the expression of several common immediate early genes including *Fos*, *Fosb*, *Dusp1*, *Nr4a1*, *Arc* and *Egr1* in integrated microglia clusters (Fig. 1b).

Figure S5

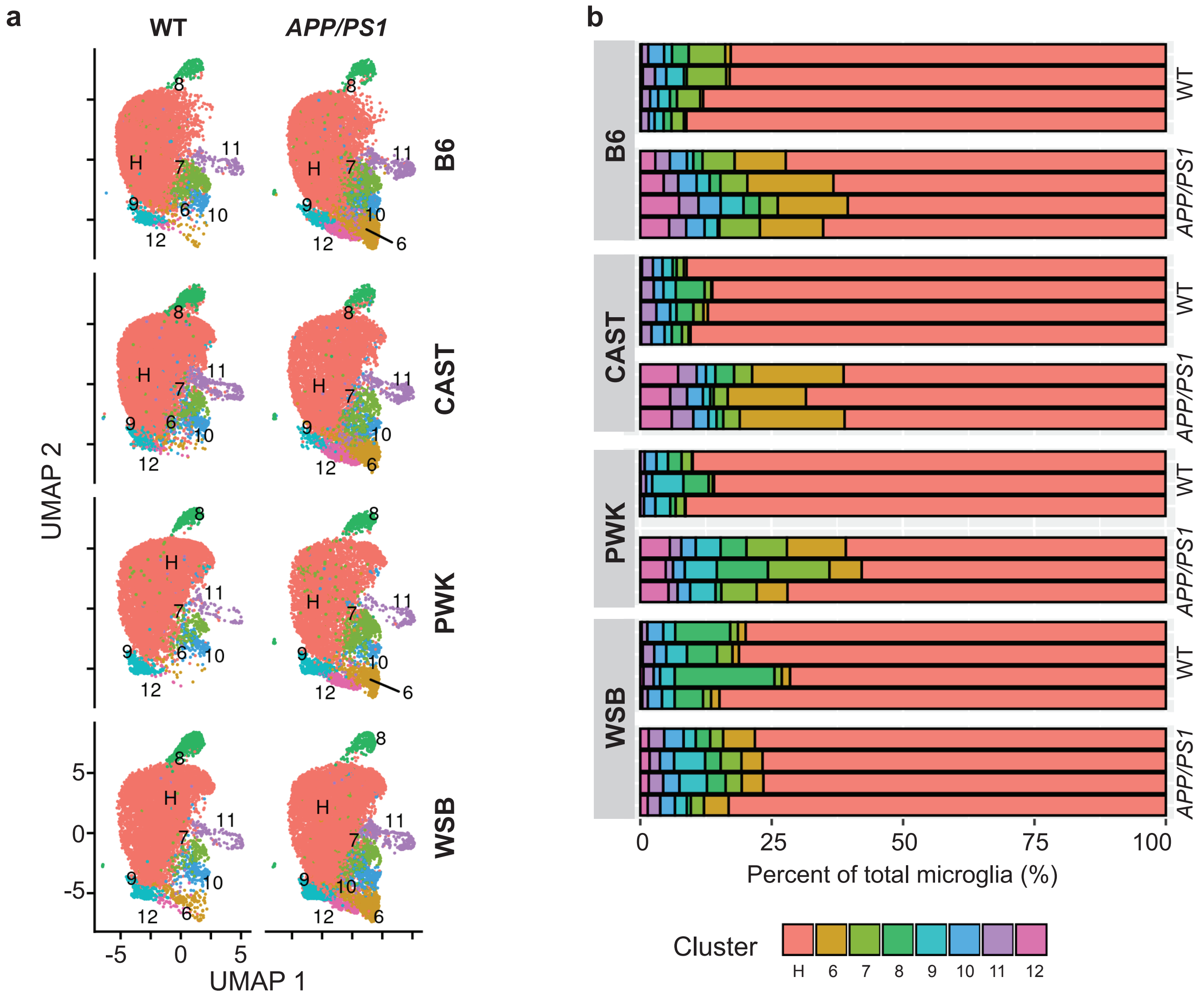

Fig. S5 | Variation in the percent of microglia subtype across strain and genotype, Related to Fig.2. **a**, UMAP plot showing microglia clusters in each strain and genotype. **b**, The percent of 8 microglia subtypes in each replicate of each strain and genotype.

Figure S6

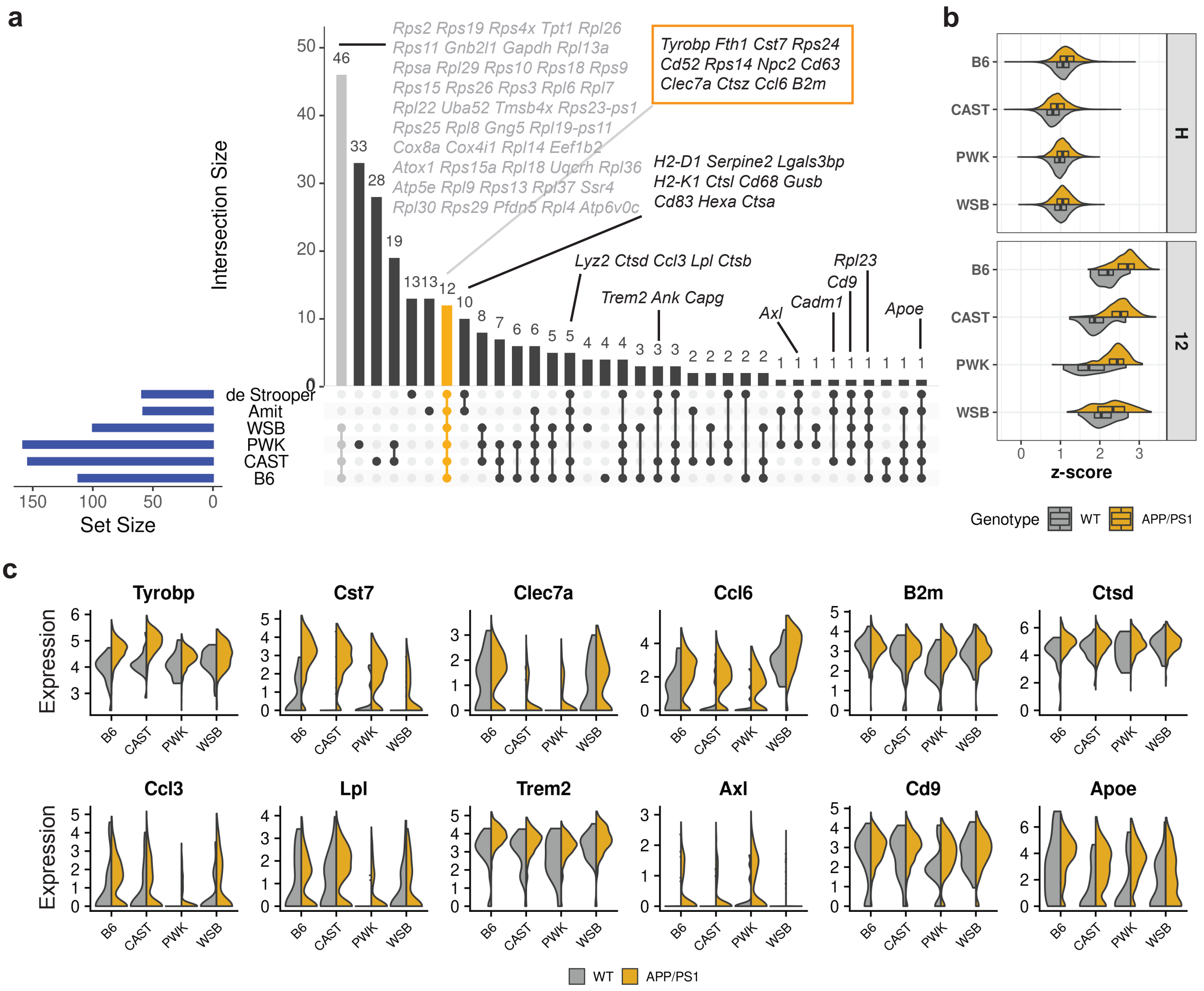

Fig. S6 | Strain-specific gene expression in disease associated microglia (cluster 12), Related to Fig.4. a, Upset plot illustrating the intersection of the top marker genes for cluster 12 in B6 and wild-derived strains integrated with top DAM marker genes from the Amit study[3] and top ARM marker genes from the de Strooper study[8]. The genes in selected intersections are detailed in the plot. Core genes shared by all datasets are highlighted in the orange box; genes shared in only between B6 and wild-derived strains from our study are colored in grey. **b**, Violin-box plots showing the enrichment z-score of core marker genes in cluster H (homeostatic) and cluster 12 for each strain and genotype. Significant strain and genotype effects were detected for each cluster ($p \approx 0$, two-way ANOVA). **c**, Violin plots showing the expression of selected marker genes in cluster 12 in each strain and genotype.

Figure S7

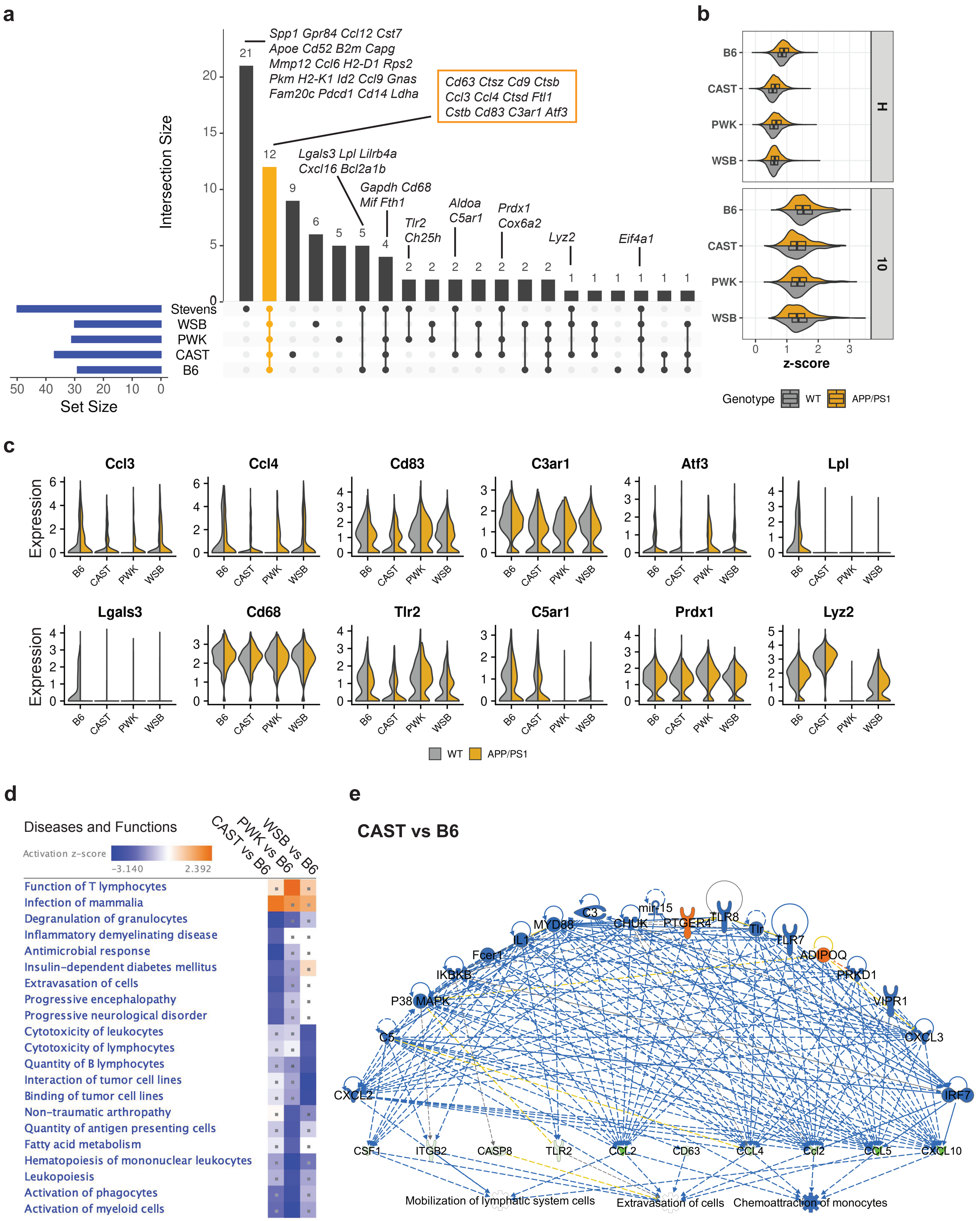
a
Intersection Size
Spp1 Gpr84 Ccl12 Cst7
Apoe Cd52 B2m Capg
Mmp12 Ccl6 H2-D1 Rps2
Pkm H2-K1 Id2 Ccl9 Gnas
Fam20c Pdcd1 Cd14 Ldha
Cd63 Ctsz Cd9 Ctsb
Ccl3 Ccl4 Ctsd Ftl1
Cstb Cd83 C3ar1 Atf3
Lgals3 Lpl Lilrb4a
Cxcl16 Bcl2a1b
Gapdh Cd68
Mif Fth1
Tlr2
Ch25h
Aldoa
C5ar1
Prdx1
Cox6a2
Lyz2
Eif4a1
Stevens
WSB
PWK
CAST
B6
Set Size
b
H
10
z-score
Genotype
WT
APP/PS1
c
Ccl3
Ccl4
Cd83
C3ar1
Atf3
Lpl
Lgals3
Cd68
Tlr2
C5ar1
Prdx1
Lyz2
Expression
d
Diseases and Functions
Activation z-score
-3.140
2.392
CAST vs B6
PWK vs B6
WSB vs B6
Function of T lymphocytes
Infection of mammalia
Degranulation of granulocytes
Inflammatory demyelinating disease
Antimicrobial response
Insulin-dependent diabetes mellitus
Extravasation of cells
Progressive encephalopathy
Progressive neurological disorder
Cytotoxicity of leukocytes
Cytotoxicity of lymphocytes
Quantity of B lymphocytes
Interaction of tumor cell lines
Binding of tumor cell lines
Non-traumatic arthropathy
Quantity of antigen presenting cells
Fatty acid metabolism
Hematopoiesis of mononuclear leukocytes
Leukopoiesis
Activation of phagocytes
Activation of myeloid cells
e
CAST vs B6
MYD88
C3
mir-15
CHUK
PTGER4
TLR8
Tlr
TLR7
ADIPOQ
PRKD1
VIPR1
IL1
Fcer1
IKBKB
P38 MAPK
C5
CXCL3
CXCL2
IRF7
CSF1
ITGB2
CASP8
TLR2
CCL2
CD63
CCL4
Ccl2
CCL5
CXCL10
Mobilization of lymphatic system cells
Extravasation of cells
Chemoattraction of monocytes

Fig. S7 | Strain-specific gene expression in *Ccl3*^{high}/*Ccl4*^{high} microglia (cluster 10), Related to Fig.2. a, Upset plot illustrating the intersection of the top *Ccl3*^{high}/*Ccl4*^{high} marker genes in B6 and wild-derived strains integrated with top marker genes in Aging OA2 cluster from the Stevens study[15]. The genes in selected intersections were detailed in the plot. Core genes shared by all datasets were highlighted in the orange box; genes only shared between B6 and wild-derived strains in our study were colored in grey. **b**, Violin-box plots showing the enrichment z-score of core *Ccl3*^{high}/*Ccl4*^{high} marker genes in cluster H (homeostatic) and cluster 10 for each strain and genotype. Significant strain and genotype effects were detected for each cluster ($p \approx 0$, two-way ANOVA). **c**, Violin plots showing the expression of selected marker genes for each strain and genotype. **d**, Heat map summarizing top 21 significantly enriched Diseases and Functions terms based on DE genes from comparisons of wild-derived vs B6 mice (pval-BH < 0.05, |z-score| ≥ 2). The dot indicates the enrichment of Diseases and Functions term was not significant for a given comparison (pval-BH ≥ 0.05). **e**, Selected Regulatory Effects for CAST vs B6 highlighting network of 'Mobilization of lymphatic system cells', 'Extravasation of cells' and 'Chemoattraction of monocytes'. The color code is the same as described in Fig. 3d-e.
